# Adherence to post-cardiac arrest care guidelines and impact on survival and neurological outcome

**DOI:** 10.1186/s13613-025-01508-1

**Published:** 2025-07-02

**Authors:** Giulia Merigo, Fabiana Madotto, Aurora Magliocca, Gaetano Florio, Alessandra Rosati, Valentina Castagna, Marco Pagliano, Alberto Zanella, Mauro Panigada, Giacomo Grasselli, Giuseppe Ristagno

**Affiliations:** 1 Department of Pathophysiology and Transplantation, University of Milan, Milan, Italy; 2 Department of Anesthesiology, Intensive Care and Emergency, Fondazione IRCCS Ca’ Granda Ospedale Maggiore Policlinico, Milan, Italy; 3 University of Milan, Milan, Italy

**Keywords:** Cardiac arrest, Post-resuscitation care, Guidelines, Survival, Neurological outcome

## Abstract

**Background:**

Post-cardiac arrest (CA) care guidelines (GLs) have been introduced in 2010 and periodically updated every 5 years since then (in 2015 and 2021). However, the impact of these GLs on patients’ outcome remains underexplored. The aim of this study was to comprehensively evaluate and compare the impact of implementation of three consecutive post-CA GLs over 14 years, on patients’ survival and neurological recovery.

**Methods:**

This retrospective cohort study included adult patients resuscitated from CA and admitted to the intensive care unit (ICU) between 2011 and 2024. Patients were stratified into three cohorts based on the GL in use (GL2010, GL2015, and GL2024). Adherence to GL recommendations was assessed across seven macro-areas: coronary angiography, haemodynamic, ventilation, temperature control, general ICU management, multimodal neuroprognostication, and seizure control. Predictors of survival and favourable neurological outcome at ICU discharge were evaluated using multivariate logistic regression with LASSO selection. Outcome up to 6 months was also evaluated.

**Results:**

A total of 275 patients were included over the 14-year period. Survival to ICU discharge increased from 39.5% in cohort 1 to 53.9% in cohort 3, together with favourable neurological outcome that improved from 30.9 to 42.7%. Adherence to GL recommendations significantly improved across most domains, particularly in haemodynamic management (from 32.0% in cohort 1 to 77.3% in cohort 3), temperature control (from 60.6 to 94.4%), and general ICU management (from 56.3 to 77.6%). Among all interventions, adherence to haemodynamic recommendations was independently associated with improved survival (OR = 2.20, 95% CI: 1.01–4.86).

**Conclusions:**

Following the implementation of updated post-CA care GLs, adherence to recommendations improved, particularly in haemodynamic management. Although no statistically significant improvements in survival or neurological outcomes were observed, these findings highlight the potential value of sustained GL-based care.

**Supplementary Information:**

The online version contains supplementary material available at 10.1186/s13613-025-01508-1.

## Background

Cardiac arrest (CA) is the third leading cause of death in Europe, representing a significant public health challenge with high mortality rates and significant long-term sequelae in survivors [1, 2]. A growing body of evidence emphasizes the pivotal role of post-CA management, a critical link in the chain of survival, underscoring its importance to improve patient outcomes [3, 4]. Thus, starting from 2010, the European Resuscitation Council (ERC) first introduced a post-resuscitation care section within the advanced life support (ALS) chapter, and updated recommendations every 5 years to incorporate emerging evidence and optimize treatment strategies [5–7]. More specifically in 2015, ERC in collaboration with the European Society of Intensive Care Medicine (ESICM) formally introduced a new guideline (GL) chapter named “post-CA care”, emphasizing the need for urgent post-resuscitation coronary angiography (CAG) and percutaneous coronary intervention (PCI) for CA from a suspected cardiac cause, the importance of a target temperature management (TTM) between 32 and 36 °C, and the adoption of a multimodal neuroprognostication strategy [7, 8]. In 2021, the post-CA care GLs were updated, including among the numerous novelties, a new temperature control (TC) strategy, targeting fever prevention (i.e. >37.7 °C), recommendations for emergent CAG in patients with high-risk features, and an overall greater emphasis on general intensive care unit (ICU) patient management with a refined neuroprognostication algorithm [6, 9]. 

Despite these efforts, evidence on the impact of introduction and implementation of a new post-CA care GL every 5 years on survival and neurological recovery remains underexplored. Indeed, available outcome studies primarily focused on adherence to pre-hospital resuscitation protocols, with post-CA data mainly limited to the early phase after return of spontaneous circulation (ROSC) [10–14]. Furthermore, most research on CA patients who were resuscitated and admitted to the ICU has focused on specific aspects of post-CA care, often neglecting a comprehensive evaluation across multiple treatment domains.

This study aimed to comprehensively evaluate and compare the impact of implementation of three consecutive post-CA care GLs, i.e. 2010, 2015, and 2021, on survival and neurological outcome in CA patients admitted to ICU.

## Methods

### Study design and setting

This retrospective, observational cohort study included all adult patients with out-of-hospital and in-hospital CA who were admitted to the ICU of the IRCCS Fondazione Ca’ Granda Ospedale Maggiore Policlinico in Milan (Italy), over a 14-year period (January 1st, 2011– September 30th, 2024). The study complies with the Declaration of Helsinki and was approved by the Regional Ethics Committee “Lombardia 3” (approval no. OSMAMI-25/07/2024-0029769-U) and registered on ClinicalTrials.gov (NCT06608771). Written informed consent was waived in accordance with local regulations on retrospective study design.

### Eligibility criteria

**Fig. 1:**
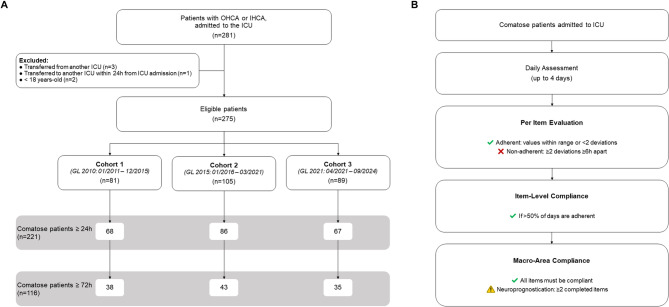
Flowcharts of the study population and of adherence assessment. **Panel A** shows the selection process of the study population, including inclusion and exclusion criteria. **Panel B** shows the stepwise evaluation of adherence. Adherence was defined as clinical values staying within the recommended range. or deviating only once (i.e., not more than one consecutive out-of-range measurement). Non-adherence was defined when two or more consecutive values (taken at least six hours apart) fell outside the recommended range. This evaluation was performed daily for each item of guidelines, until four days after ICU admission. An item was considered overall compliant if adherence was observed on more than half of the monitored days. A macro-area was considered adherent to guidelines if all its items met the compliance criteria above. The only exception was the multimodal neuroprognostication macro-area, where adherence required at least two neurological items to be completed, in accordance with GL recommendations

All adult patients (> 18 years) who suffered a CA (either out-of-hospital or in-hospital) and survived to be admitted to the ICU, were evaluated. To assess post-CA care and adherence to GL recommendations, only patients who remained comatose for at least 24 h following ICU admission were included in the analyses. Exclusion criteria included patients with age < 18 years old, and patients transferred from another ICU at any time or transferred to another ICU within the first 24 h (Fig. 1**-panel A**).

### Objectives and endpoints

The primary objective of this study was to evaluate the impact of the implementation of three consecutive post-CA care guidelines (GL2010, GL2015, and GL2021) on ICU survival and favourable neurological outcome at ICU discharge. Secondary objectives included describing the trend in adherence to guideline recommendations across seven post-CA care domains (coronary angiography, haemodynamic management, ventilation, temperature control, general ICU management, multimodal neuroprognostication, and seizure control) and identifying predictors of ICU survival and neurological recovery.

Primary endpoints were survival to ICU discharge and neurological outcome at ICU discharge (with Cerebral Performance Category (CPC) 1–2 defined as a good neurological outcome and CPC 3–5 as a poor outcome). Secondary endpoints included hospital survival, survival at 1 and 6 months, ICU and hospital length of stay (LOS), and rate of withdrawal of life-sustaining therapy (WLST).

### Data collection

The study population was stratified into three time-based cohorts according to the active GL at the time of ICU admission: cohort 1 (GL 2010, January 1st, 2011– December 31st, 2015), cohort 2 (GL 2015, January 1st, 2016– March 31st, 2021), and cohort 3 (GL 2021, April 1st, 2021– September 30th, 2024). Patients’ medical record data were retrieved by two independent researchers (GM and VC) with supervision by a senior researcher (GF) from the electronic chart DIGISTAT (version 4.3.3, 2017, Ascom, Florence, Italy) in use in the ICU.

We collected the following variables during the period up to 96 h after ICU admission:

- Patient demographics, anamnestic information (comorbidities and active drug treatments) and CA/cardiopulmonary resuscitation (CPR) characteristics (i.e., presenting CA rhythm, witnessed, lay bystander CPR, no-flow and low-flow duration, epinephrine dose, amiodarone use, defibrillation attempts, use of mechanical chest compression device - LUCAS^®^ Chest Compression System (Stryker/Jolife AB, Lund, Physio-Control, Sweden)).
- Respiratory and ventilation data: arterial oxygen pressure (PaO_2_), arterial carbon dioxide pressure (PaCO_2_), arterial oxygen saturation (SaO_2_), tidal volume (TV), positive end-expiratory pressure (PEEP) level, fraction of inspired oxygen (FiO_2_), and PaO_2_/FiO_2_.
- Cardiovascular care: cardiac aetiologies, diagnosis of ST elevation myocardial infarction (STEMI), CAG and PCI, use of antiarrhythmic medications, blood pressure control, daily urinary output, volume of IV fluid infusion, inotropes/vasopressors use.
- Metabolic data: arterial glucose and lactate.
- Neurological care: TTM (assessed with non-invasive methods), multimodal neurological assessment (including clinical examination, neurophysiology techniques, biomarkers evaluation and neuroimaging) and seizure control (based on electroencephalographic results confirmed by neurologist consultation).
- Clinical outcomes: CPC score at ICU discharge, survival at ICU and hospital discharge, WLST, ICU and hospital LOS, survival at 1 and 6 months.

### Evaluation of post-CA care guidelines and adherence

For each GL (2010, 2015, and 2021), seven macro-areas were defined for the adherence evaluation: coronary angiography, haemodynamic, ventilation, temperature management, general ICU management, multimodal neuroprognostication assessment, and seizure control. As reported in Table 1, each macro-area included specific items aligned with GL recommendations: [4, 6–9]

**Table 1:** Guideline macro-areas and corresponding items aligned with recommendations

Item	GL 2010 recommendations	GL 2015 recommendations	GL 2021 recommendations
**Macro-area– Coronary Angiography**			
CAG	Reasonable to perform immediate angiography and PCI in selected patients, despite the absence of ST-elevation on the ECG or prior clinical findings such as chest pain	Consider emergent cardiac characterisation laboratory evaluation after ROSC in patients with the highest risk of a coronary cause for their CA	Emergent cardiac catheterisation laboratory evaluation (and immediate PCI if required) should be per formed in adult patients with ROSC after CA of suspected cardiac origin with ST-elevation on the ECG.
**Macro-area– Haemodynamic**			
MAP	*na*	Avoid hypotension (MAP < 65 mmHg)	Avoid hypotension (MAP < 65 mmHg)
Urinary output	Target the MAP to achieve adequate urine output (1 ml/kg/h)	Target the MAP to achieve adequate urine output (1 ml/kg/h)	Target the MAP to achieve adequate urine output (> 0.5 ml/kg/h)
Lactate clearance	Normal or decreasing plasma lactate	Normal or decreasing plasma lactate	Normal or decreasing plasma lactate
**Macro-area– Ventilation**			
SaO_2_	≥ 94%	≥ 94%	≥ 94%
PaO_2_	*na*	75–100 mmHg (10–13 kPa)	75–100 mmHg (10–13 kPa)
PaCO_2_	*na*	35–45 mmHg (4.5–6.0 kPa)	35–45 mmHg (4.5–6.0 kPa)
TV	*na*	6–8 ml/kg	6–8 ml/kg
PEEP	*na*	4–8 cmH_2_O	5–10 cmH_2_O
**Macro-area– Temperature Management**			
TTM	32–34 °C for 12–24 h	32–36 °C for at least 24 h	Avoid fever (> 37.7 °C) for at least 72 h
**Macro-area– General ICU Management**			
Blood glucose control	Maintain blood glucose at ≤ 180 mg/dl, and avoid hypoglycaemia (< 70 mg/dl)	Maintain blood glucose at ≤ 180 mg/dl, and avoid hypoglycaemia (< 70 mg/dl)	Maintain blood glucose at ≤ 180 mg/dl, and avoid hypoglycaemia (< 70 mg/dl)
DVTP	*na*	*na*	Provide DVTP
SUP	*na*	*na*	Provide SUP
**Macro-area– Multimodal Neuroprognostication**			
Pupillometry	*na*	*na*	Quantitative pupillometry
Pupillary and corneal reflexes	*na*	Evaluation of pupillary and corneal reflexes	Evaluation of pupillary and corneal reflexes
EEG	*na*	Perform EEG	Perform EEG
SSEP	*na*	Perform SSEP	Perform SSEP
Biomarkers evaluation	*na*	NSE dosage	NSE dosage
CT or MRI imaging	*na*	Brain imaging studies	Brain imaging studies
**Macro-area– Seizure Control**			
Seizure detection and treatment, including AED administration	*na*	Seizure detection and treatment, including AED administration	Seizure detection and treatment, including AED administration

Note. If the guideline in effect during the period did not include the item, adherence was considered as “not assessable” (na)

Abbreviations. AED, antiepileptic drugs; CAG, coronary angiography; CT, computed tomography; DVTP, deep venous thrombosis prophylaxis; EEG, electroencephalogram; ICU, intensive care unit; MAP, mean arterial pressure; MRI, magnetic resonance imaging; NSE, neurological status evaluation; PaO_2_, arterial oxygen pressure; PaCO_2_, arterial carbon dioxide pressure; PCI, percutaneous coronary intervention; PEEP, positive end expiratory pressure; ROSC, return of spontaneous circulation; SaO_2_, arterial oxygen saturation; SSEP, somatosensory evoked potential; SUP, stress ulcer prophylaxis; TTM, targeted temperature management; TV, tidal volume

- Coronary angiography.
- Haemodynamics: mean arterial pressure (MAP), urinary output, lactate clearance.
- Ventilation: SaO_2_, PaO_2_, PaCO_2_, TV, PEEP.
- Temperature management.
- General ICU management: blood glucose control, deep venous thrombosis prophylaxis (DVTP), stress ulcer prophylaxis (SUP).
- Multimodal neuroprognostication assessment: quantitative pupillometry, pupillary and corneal reflexes, somatosensory evoked potentials (SSEP), electroencephalogram (EEG), status myoclonus during the first 72 h, biomarkers evaluation (neuron serum enolase (NSE)), neuroimaging (computed tomography (CT) and magnetic resonance imaging (MRI)).
- Seizure control.

The assessment of GL adherence was based on the execution of the recommended procedures/interventions and the monitoring of patient’s clinical parameters during the first 96 h of ICU stay. With the exception of coronary angiography procedure, which was performed before ICU admission, all other procedures (DVTP, SUP, pupillometry, reflexes, SSEP, EEG, NSE, neuro imaging, and seizure treatment) were recorded daily, along with all clinical parameters (MAP, urinary output, lactate clearance, TTM, SaO_2_, PaO_2_, PaCO_2_, TV, PEEP, blood glucose) recorded at regular intervals (not exceeding 6 h) within this period. Adherence was evaluated based on the consistency between observed clinical values and guideline-recommended targets, regardless of whether a specific intervention was explicitly prescribed or documented.

For each item, adherence to GL was evaluated daily, based on the recommendations summarized in Table 1. Adherence was defined as clinical measurements remaining within the recommended range, or deviating for less than two consecutive assessments. Non-adherence was defined when two or more consecutive measurements, taken at least 6 h apart, were outside the recommended range. These definitions were applied to each item on a daily basis, for up to four days following ICU admission. Finally, overall compliance for each item was defined as adherence being present on more than 50% of the observation days.

A macro-area was, instead, considered adherent to GL if all its items were compliant to recommendations (as defined above), except for the multimodal neuroprognostication macro-area, where the completion of at least two neurological items were considered as necessary (as per GL recommendations). Additional details on the adherence definition are provided in the Supplementary Appendix and Fig. 1 (panel B).

Of note, in accordance with GL indications, multimodal neuroprognostication assessment was performed exclusively on comatose patients, who remained unresponsive for at least 72–96 h after ROSC. Moreover, data collected during the WLST period and/or on the day of the patient’s death were not included for the adherence detection.

### Statistical analysis

Considering three cohorts of post-CA patients admitted to the ICU, the study aimed to test whether there was a positive linear trend in the proportion of survivors at ICU discharge (H0: p1 = p2 = p3 vs. H1: p1 < p2 < p3), with a Type I error probability of 5% (alpha) and a power of 80% (1-beta). Assuming survival rates of 25% (p1), 35% (p2), and 45% (p3) in the three cohorts, the study planned to enrol at least 70 patients in each cohort (for a total of 210 patients) to test the hypothesis using the Cochran–Armitage trend test.

According to data distribution, continuous variables were reported as mean and standard deviation (SD) or as median and interquartile range (first and third quartiles) (IQR). Categorical variables are expressed as counts and percentages. No imputation procedures were used for missing values. Number of patients with missing data for each variable of interest was reported in Supplementary Tables S1. For categorical variables, comparisons among the 3 cohorts were performed using the Chi-square test or Fisher’s exact test, as appropriate. The Cochran-Armitage trend test was used to assess linear trends in the proportions of categorical variables across cohorts. For continuous variables, one-way Analysis of Variance (ANOVA) was used when the variable was normally distributed, as assessed by the Shapiro-Wilk test. For continuous variables not Normally distributed, the Kruskal-Wallis test was applied, and if necessary, the variables were transformed using the Box-Cox transformation to achieve normality. For post-hoc pairwise comparisons between cohorts, Dunnett’s test was used, with the cohort 1 as the reference group.

To investigate the factors influencing survival, the following variables were considered as potential predictors for ICU survival: cohort, adherence to the macro-areas of post-CA care GL (haemodynamic, ventilation, TTM and ICU management), sex, out-of-hospital CA (OHCA), cardiac aetiology, shockable rhythm, lay bystander CPR, mechanical chest compression, epinephrine dose, comorbidities, drug treatment, age, no-flow time, low-flow time, Sequential Organ Failure Assessment (SOFA) score, Charlson Comorbidity Index (CCI) and GCS at hospital admission. The cohort was considered as a predictor in order to represent variations in outcomes among patients admitted during different guideline periods (GL2010, GL2015, and GL2021), likely due to changes in clinical practices, advancements in treatment, or improvements in post-cardiac arrest care over time (cohort effect). The effect of adherence to each single item within the macro-areas was also investigated.

Separate univariate logistic regression models with survival at ICU discharge and survival at ICU discharge with favourable neurological outcome as the dependent variables, were performed. All continuous variables were standardized in order to compare predictors with different units of measurement (effect sizes on the same scale). Following the univariate analysis, we applied the Least Absolute Shrinkage and Selection Operator (LASSO) using logistic regression to identify the most significant predictors for inclusion in a multivariable logistic regression model minimizing multicollinearity and overfitting. Variables selected by LASSO were subsequently entered into unpenalized multivariable logistic regression models, to estimate the odds ratios (OR) of the identified predictors, allowing for an unbiased estimation of the relationship between the predictors and the outcomes (magnitude and direction), without the influence of regularization.

All statistical tests were two-sided with a significance α-level of 0.05. Analyses were performed using R 4.3.2 (R Foundation for Statistical Computing, Vienna, Austria) and SAS 9.4 (SAS Institute, Cary, North Carolina, USA).

Additional information on the statistical analysis are provided in the Supplementary Appendix.

## Results

### Study population and CA/CPR characteristics

A total of 275 patients were included: 81 (29.45%) in cohort 1, 105 (38.16%) in cohort 2, and 89 (32.36%) in cohort 3. The study population and CA/CPR characteristics are summarized in Table 2, with further details on CA characteristics, comorbidities, and active drug treatments provided in Supplementary Tables S2-S4, respectively.

**Table 2:** Demographic, anamnestic, and cardiac arrest/resuscitation characteristics of the study population, stratified by cohort

	All patients	Cohort 1 GL 2010 (01/2011–12/2015)	Cohort 2 GL 2015 (01/2016–03/2021)	Cohort 3 GL 2021 (04/2021–09/2024)	*p*-value
**Demographic characteristics**					
Patients, n (%)	275 (100.00)	81 (29.45)	105 (38.16)	89 (32.36)	-
Male sex^§^, n (%)	207 (75.27)	55 (67.90)	76 (72.38)	76 (85.39) ^†^	0.0209
Age (years), median [IQR]	61.00 [51.00–70.00]	65.00 [55.00–73.00]	61.00 [50.00–71.00]	59.00 [51.00–67.00]	0.0770
CCI (score), median [IQR]	3.00 [1.00–5.00]	3.00 [2.00–5.00]	3.00 [2.00–5.00]	2.00 [1.00–4.00] ^†^	0.0201
**CA and CPR characteristics**					
OHCA	178 (66.67)	54 (66.67)	60 (57.14)	64 (71.91)	0.0912
Pathogenesis					
Medical	234 (85.09)	71 (87.65)	86 (81.90)	77 (86.52)	0.4960
Cardiac aetiology	163 (69.96)	50 (71.43)	51 (59.30)	62 (80.52)	0.0122
Traumatic	9 (3.27)	2 (2.47)	4 (3.81)	3 (3.37)	0.9164
Drug overdose	14 (5.09)	0 (0.00)	10 (9.52) ^†^	4 (4.49)	0.0064
Drowning	0 (0.00)	0 (0.00)	0 (0.00)	0 (0.00)	-
Electrocution	0 (0.00)	0 (0.00)	0 (0.00)	0 (0.00)	-
Asphyxia	18 (6.55)	8 (9.88)	5 (4.76)	5 (5.62)	0.3429
Shockable rhythm ^§^, n (%)	122 (44.53)	31 (38.27)	40 (38.46)	51 (57.30) ^†^	0.0128
Non-shockable rhythm ^§^, n (%)	122 (44.53)	50 (61.73)	64 (61.54)	38 (42.70) ^†^	0. 0128
Witnessed cardiac arrest, n (%)	252 (91.64)	73 (90.12)	99 (94.29)	80 (89.89)	0.4584
Lay Bystander CPR, n (%)	101 (36.73)	27 (33.33)	34 (32.38)	40 (44.94)	0.1466
No flow (min), median [IQR]	0.00 [0.00–3.00]	0.00 [0.00–0.00]	0.00 [0.00–5.00]	0.00 [0.00–3.00]	0.0711
Low flow (min), median [IQR]	18.00 [8.00–30.00]	17.00 [9.00–25.00]	15.00 [6.00–30.00]	21.00 [12.00–34.00]	0.1391
Mechanical CC ^§^, n (%)	76 (27.64)	2 (2.47)	45 (42.86) ^†^	29 (32.58) ^†^	< 0.0001
Epinephrine dose (mg), median [IQR]	2.00 [1.00–4.00]	2.00 [1.00–4.00]	2.00 [1.00–4.00]	2.00 [1.00-4.50]	0.6607
Amiodarone use ^§^, n (%)	74 (27.01)	18 (22.50)	16 (15.24)	40 (44.94) ^†^	< 0.0001
Defibrillation attempts, median [IQR]	1.00 [0.00–3.00]	0.00 [0.00–3.00]	0.00 [0.00–2.00]	2.00 [0.00–3.00] ^†^	0.0058
Patients with comorbidities, n (%)	233 (84.73)	66 (81.48)	90 (85.71)	77 (86.52)	0.6192
Patients with active drug treatment, n (%)	160 (58.18)	41 (50.62)	70 (66.67)	49 (55.06)	0.0682
SOFA score, median [IQR]	9.00 [7.00–11.00]	9.00 [7.00–11.00]	9.00 [7.00–11.00]	9.00 [8.00–11.00]	0.9048

Abbreviations. CA, cardiac arrest; CC, Chest Compression; CCI, Charlson’s comorbidity index; CPR, cardiopulmonary resuscitation; IQR, interquartile range [1st quartile– 3rd quartile]; OHCA, out-of-hospital CA; SOFA, sequential organ failure assessment. † *p* < 0.05, comparison vs. “Cohort 1” (adjusted for multiple comparison, Dunnett’s test). § *p* < 0.05, Cochran Armitage test for trend

### Post-CA management

Post-CA interventions are summarized in Table 3. Among haemodynamic support strategies, most of the population was treated with vasopressor/inotropic drugs. Despite the increasing use of norepinephrine as a first-line vasopressor, a substantial proportion of patients also received epinephrine, reflecting its use as a second-line agent in cases of persistent circulatory shock unresponsive to norepinephrine alone. Significant positive trends were also noted in most of the multimodal neuroprognostication approaches (Table 3, Table S5).

**Table 3:** Post-cardiac arrest management in the study population, stratified by cohort

	All patients (*n* = 275)	Cohort 1 GL 2010 (01/2011–12/2015) (*n* = 81)	Cohort 2 GL 2015 (01/2016–03/2021) (*n* = 105)	Cohort 3 GL 2021 (04/2021–09/2024) (*n* = 89)	*p*-value
**Treatments and procedures, ***n* (%)					
Coronary angiography	107 (38.91)	30 (37.04)	31 (29.52)	46 (51.69) ^†^	0.0063
PCI	91 (33.21)	26 (32.50)	28 (26.67)	37 (41.57)	0.0884
ECMO V-A ^§^	35 (12.73)	5 (6.17)	13 (12.38)	17 (19.10) ^†^	0.0408
ECMO V-V	2 (0.73)	1 (1.23)	1 (0.95)	0 (0.00)	0.7520
IABP	40 (14.55)	14 (17.28)	6 (5.71) ^†^	20 (22.47)	0.0031
CRRT	30 (10.91)	11 (13.58)	13 (12.38)	6 (6.74)	0.2983
Antiepileptic drugs administered	58 (21.09)	20 (24.69)	22 (20.95)	16 (17.98)	0.5626
Inotropic/vasopressor drugs administered	199 (73.16)	55 (69.62)	77 (74.04)	67 (75.28)	0.6877
Epinephrine IV use	124 (45.59)	34 (43.04)	46 (44.23)	44 (49.44)	0.6649
Norepinephrine IV use ^§^	121 (44.49)	18 (22.78)	53 (50.96) ^†^	50 (56.18) ^†^	< 0.0001
Dobutamine IV use ^§^	22 (8.09)	11 (13.92)	8 (7.69)	3 (3.37) ^†^	0.0427
Dopamine IV use ^§^	28 (10.29)	20 (25.32)	6 (5.77) ^†^	2 (2.25) ^†^	< 0.0001
**Multimodal neuroprognostication**,** n (%)**					
TTM/TC	181 (65.82)	47 (58.02)	76 (72.38)	58 (65.17)	0.1216
Pupillometry (NPI) ^§^	80 (29.41)	0 (0.00)	14 (13.46) ^†^	66 (74.16) ^†^	< 0.0001
Pupillary and corneal reflexes	222 (80.73)	59 (72.84)	88 (83.81)	75 (84.27)	0.1004
EEG	148 (53.82)	37 (45.68)	63 (60.00)	48 (53.93)	0.1515
SSEP ^§^	71 (25.91)	5 (6.17)	41 (39.42) ^†^	25 (28.09) ^†^	< 0.0001
Brain MRI ^§^	11 (4.00)	1 (1.23)	3 (2.86)	7 (7.87)	0.0945
Brain CT ^§^	191 (69.45)	47 (58.02)	67 (63.81)	77 (86.52) ^†^	< 0.0001
Patients with NSE measured ^§^	130 (47.27)	27 (33.33)	57 (54.29) ^†^	46 (51.69) ^†^	0.0107
NSE peak value (mcg/L), median [IQR]	31.25 [17.20–95.00]	30.90 [17.20–80.50]	30.10 [14.40-132.40]	33.45 [19.10–72.00]	0.9362
Patients with early myoclonus status	55 (20.00)	17 (20.99)	25 (23.81)	13 (14.61)	0.2699

Abbreviations. CRRT, continuous renal replacement therapy; CT, computed tomography; ECMO, extracorporeal membrane oxygenation; EEG, electroencephalography; IABP, intra-aortic balloon pump; IQR, interquartile range [1st quartile– 3rd quartile]; MRI, magnetic resonance imaging; NPI, neurological prognostic index; NSE, neuron serum enolase; PCI, percutaneous coronary intervention; SSEP, somatosensory evoked potentials; TTM/TC, targeted temperature management/controlled hypothermia; V-A, venous-arterial; V-V, venous-venous

† *p* < 0.05, comparison vs. “Cohort 1” (adjusted for multiple comparison, Dunnett’s test). § *p* < 0.05, Cochran Armitage test for trend

### Clinical outcomes

Patients’ clinical outcomes are detailed in Table 4. Survival to ICU discharge was 46.55% overall, increasing over time (from 39.51 to 53.93% in cohort 3, Cohran-Armitage test, *p* = 0.0589), with a similar trend observed for favourable neurological outcome (30.86% to 42.70).

**Table 4:** Clinical outcomes in the study population, stratified by cohort

	All patients (*n* = 275)	Cohort 1 GL 2010 (01/2011–12/2015) (*n* = 81)	Cohort 2 GL 2015 (01/2016–03/2021) (*n* = 105)	Cohort 3 GL 2021 (04/2021–09/2024) (*n* = 89)	*p*-value
**Primary endpoints, ***n* (%)					
ROSC	255 (99.22)	80 (100.00)	91 (100.00)	84 (97.67)	0.2072
Alive at ICU discharge	128 (46.55)	32 (39.51)	48 (45.71)	48 (53.93)	0.1658
**Secondary endpoints**,** n (%)**					
Patients with favourable neurological outcome at ICU discharge	107 (38.91)	25 (30.86)	44 (41.90)	38 (42.70)	0.2081
1-month survival ^§^	113 (41.54)	26 (32.10)	45 (42.86)	42 (48.84)	0.0848
1-month survival with favourable neurological outcome	96 (35.29)	21 (25.93)	41 (39.05)	34 (39.53)	0.1087
6-month survival	90 (35.71)	24 (31.58)	34 (35.42)	32 (40.00)	0.5461
6-month survival with favourable neurological outcome	80 (31.75)	21 (27.63)	33 (34.38)	26 (32.50)	0.6310
WLST	96 (34.91)	21 (25.93)	40 (38.10)	35 (34.91)	0.1281
**Other clinical outcomes**,** median [IQR]**					
Length of ICU stay (days)	4.00 [3.00–7.00]	4.00 [3.00–6.00]	5.00 [3.00–7.00]	4.00 [3.00–8.00]	0.8421
Length of mechanical ventilation (days)	3.00 [2.00–6.00]	3.50 [2.00–6.00]	3.00 [2.00–5.00]	3.00 [2.00–7.00]	0.7875
Length of hospital stay (days)	8.00 [4.00–18.00]	6.00 [3.00–18.00]	9.00 [4.00–17.00]	8.50 [4.00-16.50]	0.8804

Abbreviations. ICU, intensive care unit; IQR, interquartile range [1st quartile– 3rd quartile]; ROSC, return of spontaneous circulation; WLST, withholding and withdrawing life-sustaining treatment

† *p* < 0.05, comparison vs. “Cohort 1” (adjusted for multiple comparison, Dunnett’s test). § *p* < 0.05, Cochran Armitage test for trend

Clinical outcomes in the comatose patients are reported in Table S6.

### Adherence to the post-CA care guidelines

To assess the implementation and adherence to post-CA care GLs, a total of 221 comatose patients were included: 68 (30.77%) in cohort 1, 86 (38.91%) in cohort 2 and 67 (30.32%) in cohort 3 (Fig. 1). The characteristics of this comatose population, detailed in Table S7, align with those of the general population (Table 2).

**Fig. 2:**
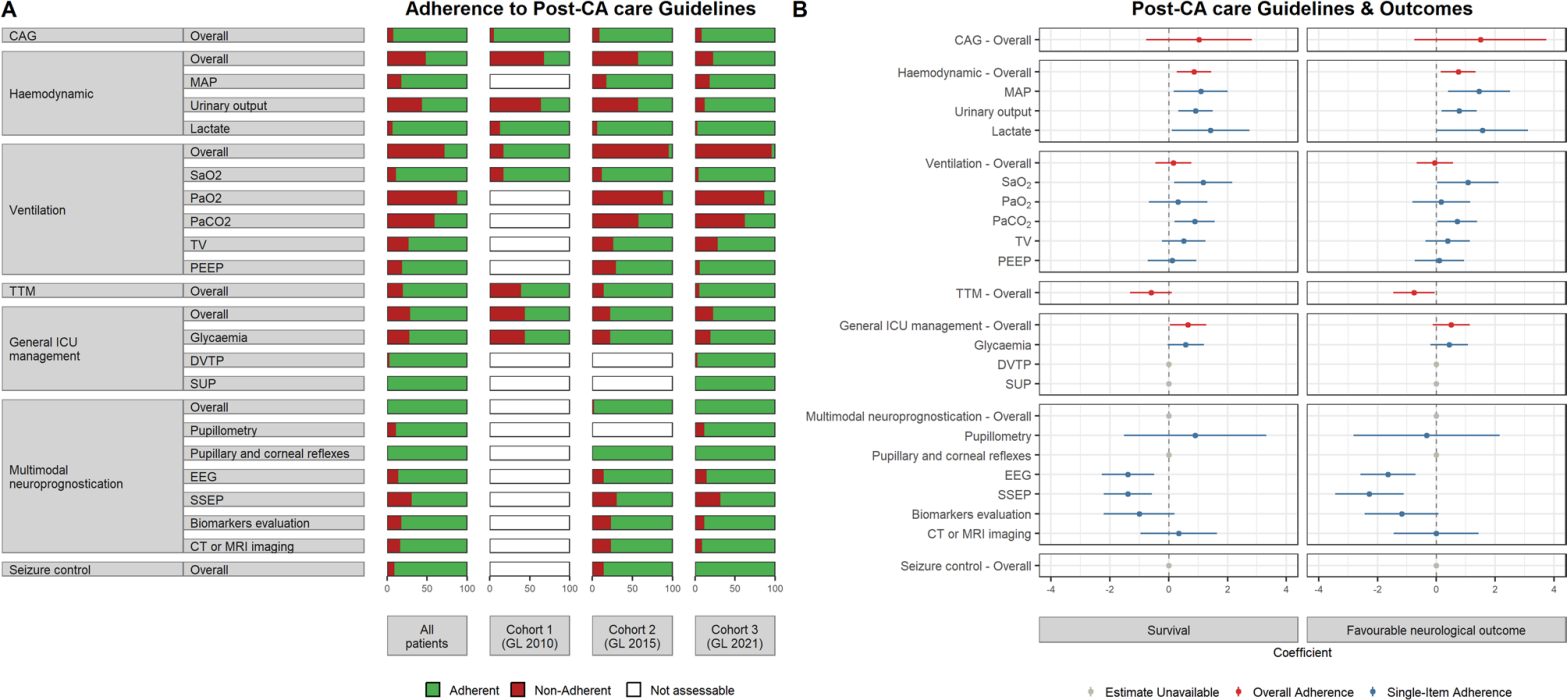
Adherence to guideline recommendations and their impact on outcomes. **Panel A** shows the percentage of patients adhering to guideline (GL) recommendations (green bar) and the percentage of non-adherent patients (red bar). The GL recommendations included 7 macro-areas: coronary angiography (CAG), hemodynamic, ventilation, targeted temperature management (TTM), general intensive care unit (ICU) management, multimodal neuroprognostication, and seizure control, each covering individual items. If specific recommendations were not included in the GL, a white bar was used in the figure to indicate non-assessable adherence. **Panel B** shows the results (beta coefficients) of univariate logistic regression models predicting ICU survival and favourable neurological outcome at ICU discharge. The beta coefficients (dots) represent the effect of adherence to each individual GL item on these outcomes. Positive coefficients indicate a favourable impact of adherence, while negative coefficients suggest an unfavourable effect. The 95% confidence intervals (horizontal lines) for the coefficients are shown, calculated using the Wald formula

Proportion of patients adhering to GL recommendations is reported in Fig. 2**(panel A)** and in Supplementary Table S8. Concerning the haemodynamics macro-area, the overall adherence was 51.50%, with a statistically significant positive trend over time (*p* < 0.0001). In the last cohort, more than 80% of patients showed adherence to each individual GL item of this macro-area. Concerning the ventilation macro-area, the overall adherence was 27.91%, with statistical differences observed among cohorts (*p* < 0.0001). The lowest adherence rates were observed for PaO_2_ and PaCO_2_ items, with 12.58% and 40.40%, respectively. Overall general ICU management recommendations were followed in 71.30% of the population, with a significant positive trend among cohorts (*p* = 0.0074). In addition, nearly all eligible patients underwent CAG. Regarding temperature management, overall adherence was 80.10%, with a significant positive trend (*p* < 0.0001). Within the context of multimodal neuroprognostication approach, the adherence was assessed in 116 patients who remained comatose for at least 72 h from ROSC; almost all these patients underwent at least two neurological examinations. Moreover, seizure detection and treatment, including specific AED administration, occurred in 90.48% of patients.

**Fig. 3:**
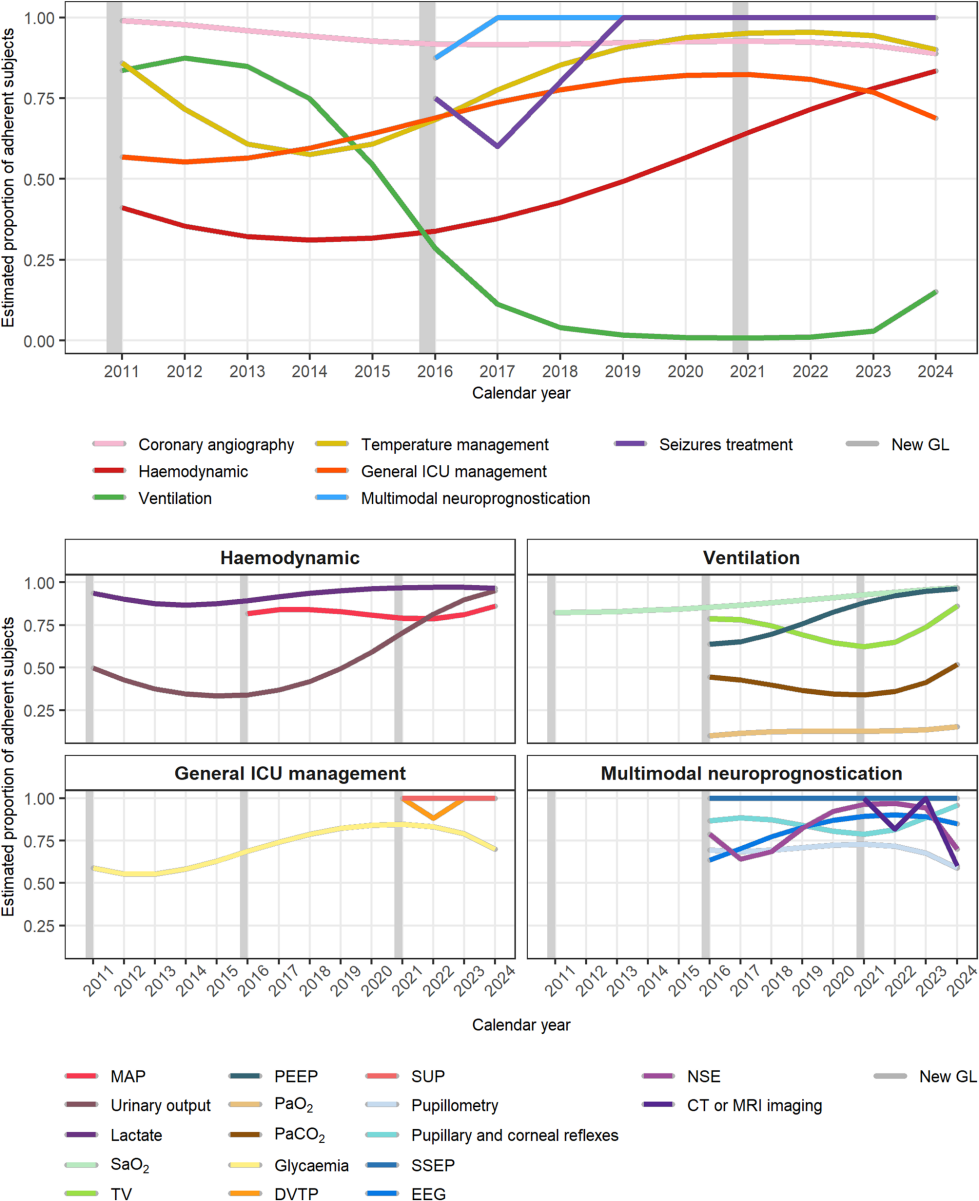
Proportion of adherent subjects over time in each macro-area and item of GL recommendations. Generalized linear model with a binomial family was applied to estimate the yearly proportion of adherent subjects

**Fig. 4:**
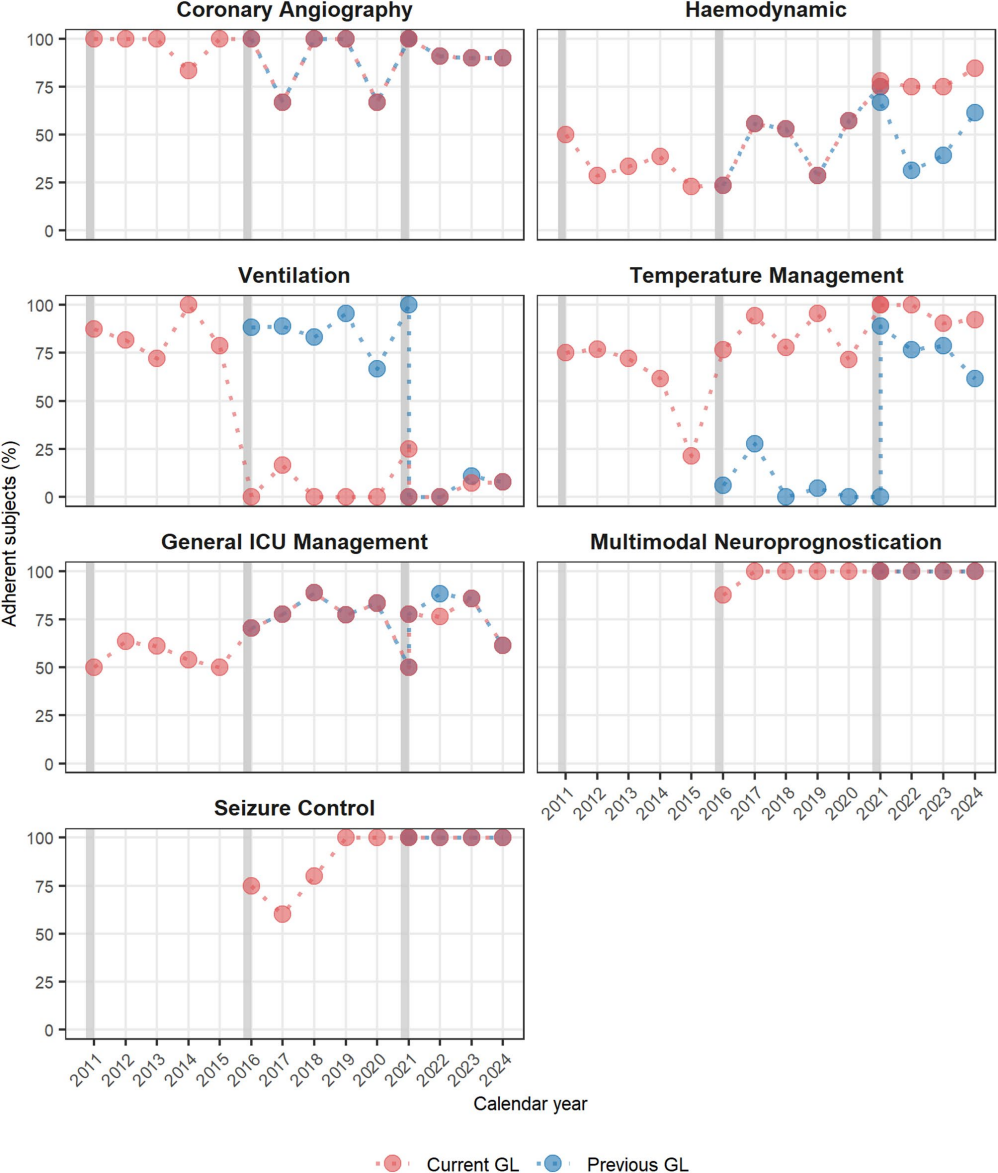
Annual trend in adherence to current and previous post-CA GL recommendations, by macro-area. For each macro-area of guideline (GL) recommendations, the figure shows the annual percentage of patients adherent to the GL in force at the time of cardiac arrest (red dots) and those who remained adherent to the previous GL (blue dots). When the recommendation remains unchanged or similar across both GLs, the two dots overlap, resulting in a purple-grey appearance. Grey vertical bands indicate the publication of a new GL. In 2021, more than two dots may appear because two GL versions were in effect, with the most recent published in March 2021

Figure 3 reports the proportion of adherent subjects over time in each macro-area and item of GL recommendations. Overall, adherence to the GL recommendations increased over time, particularly in the haemodynamic, TTM, and general ICU management macro-areas. Adherence to CAG, multimodal neuroprognostication, and seizure control remained consistently high throughout the study period. The only area that showed a decreasing trend in adherence over time was the ventilation macro-area. In most macro-areas, adherence proportions to the new GLs are similar or even higher than adherence to previous GLs already in the first year after publication (Fig. 4). An exception is the ventilation macro-area, where adherence to previous GLs remains higher due to the low percentage of patients achieving target ranges for PaO₂ and PaCO₂ (Figure S1).

### Factors associated with ICU survival

**Fig. 5:**
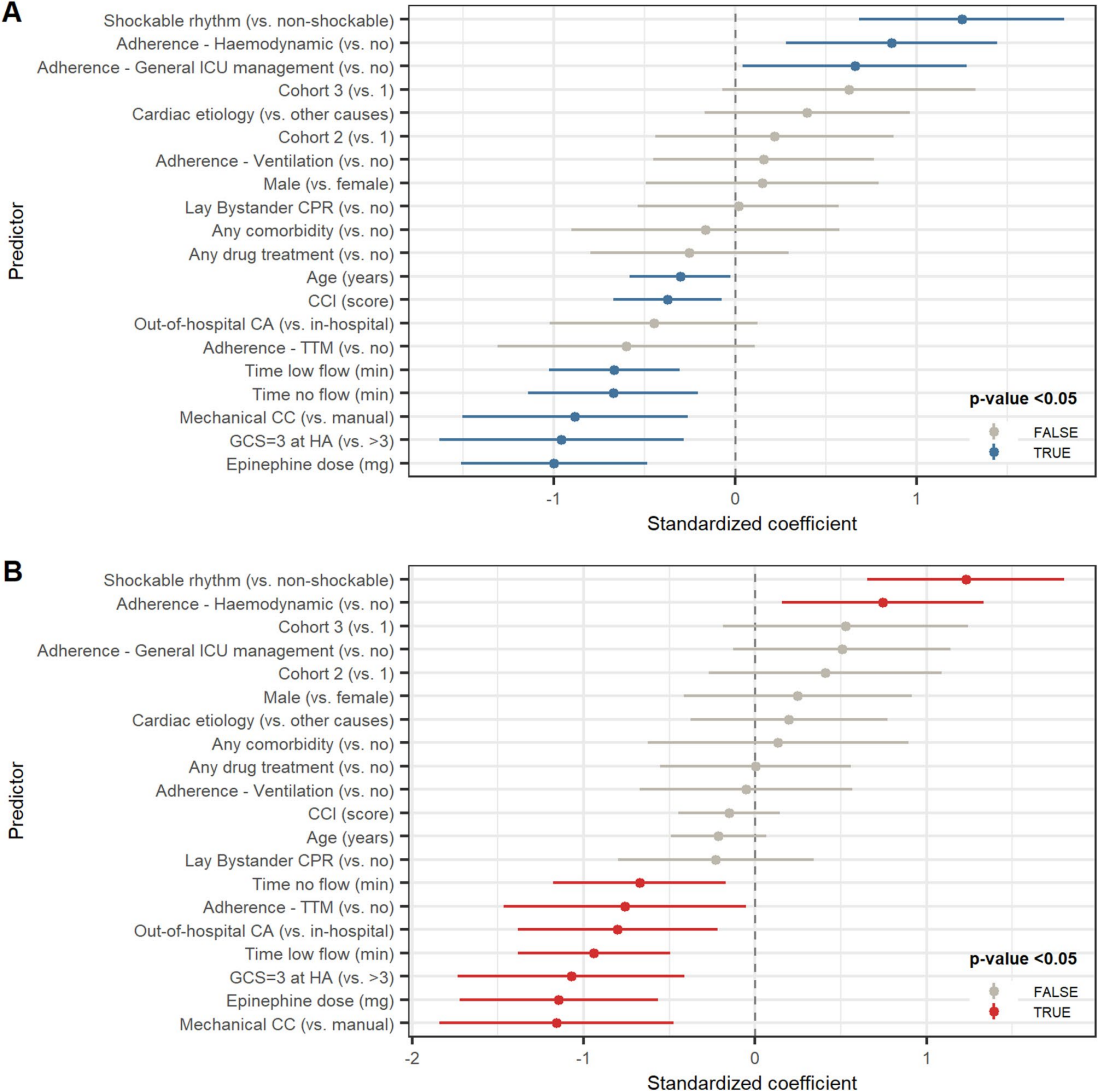
Univariate logistic regression for survival and favourable neurological outcome at ICU discharge. The beta coefficients represent the results of univariate logistic regression models predicting ICU survival (**panel a**) and favourable neurological outcome at ICU discharge (**panel b**). The 95% confidence intervals calculated using the Wald formula. Predictors with statistically significant coefficients (different from zero) are highlighted in blue/red. Note. To compare effects, continuous predictors were standardized

To assess the strength of the association between various predictors and the outcomes of interest, univariate analyses were performed. Figure 5 shows predictors of ICU survival (panel A) and survival at ICU discharge with a favourable neurological outcome (panel B). The univariate models identified several key factors associated with the outcomes of interest. Specifically, a shockable presenting rhythm and adherence to haemodynamic and general ICU management GL recommendations were positively associated with ICU survival. Conversely, older age, higher CCI, longer duration of no/low flow time, use of mechanical chest compressor device, higher epinephrine dose, and a GCS score of 3 at hospital admission were negatively associated with ICU survival. Similarly, a shockable rhythm and adherence to haemodynamic GL recommendations were positively associated with favourable neurological outcome, whereas adherence to TTM, longer duration of no/low flow time, use of mechanical chest compressor, higher epinephrine dose, and GCS score of 3 at hospital admission were negatively associated with favourable neurological outcome. Other variables showed no significant association. Concerning the effect of adherence to each item within macro-areas, Fig. 2 (panel B) reports coefficients estimated by univariate logistic models.

**Fig. 6:**
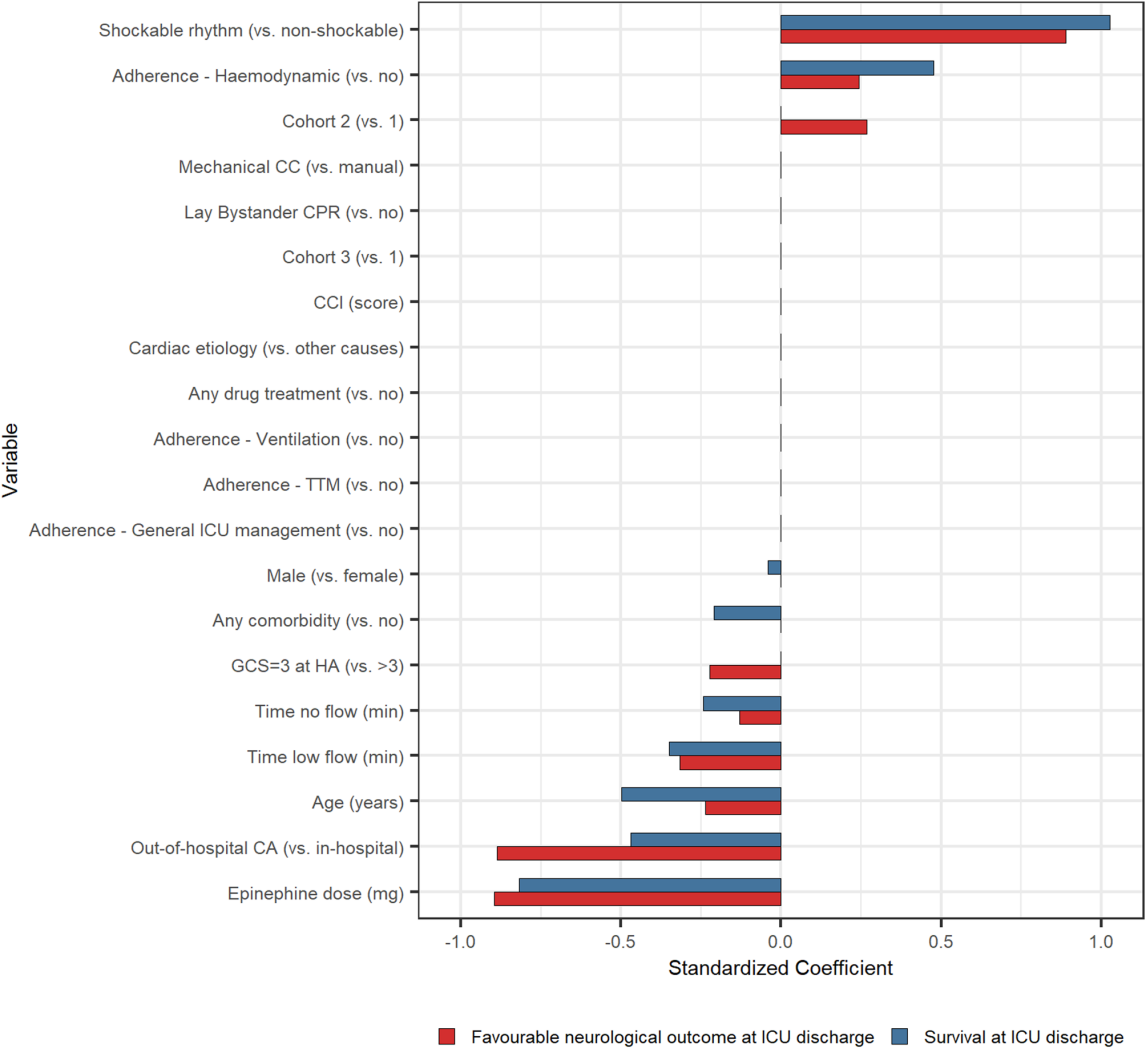
LASSO regression models for predictions of survival and favourable neurological outcome at ICU discharge. Figure shows the standardized coefficients from the LASSO logistic regression models, used to predict survival (blue bars) and favourable neurological outcome at ICU discharge (red bars). The coefficients represent the log-odds of survival or favourable neurological outcome at ICU discharge associated with a one-unit increase in each predictor variable

Following the univariate analysis, multivariate LASSO models were applied to adjust the relationship between predictors and outcomes. Figure 6 shows predictors of ICU survival and of survival at ICU discharge with favourable neurological outcome. For ICU survival, the top predictors identified were the presence of a shockable rhythm and the epinephrine dose, followed by age, OHCA, duration of no/low flow, adherence to haemodynamic GL recommendations, presence of comorbidities and gender. The accuracy of this LASSO regression model was 0.68 (95%CI: 0.49–0.83). For survival at ICU discharge with favourable neurological outcome, the top predictors identified were the presence of a shockable rhythm, the epinephrine dose and OHCA, followed by age, duration of no/low flow, adherence to haemodynamic GL recommendations, cohort effect, and GCS score of 3 at ICU admission. The accuracy of this LASSO model was 0.74 (95%CI: 0.55–0.88). The ORs from the multivariate logistic regression models were estimated to quantify the strength of association between each predictor and the outcome of interest across the entire study population (including both the training and test sets) (Table 5). For ICU survival, the presence of a shockable rhythm was associated with a significantly higher likelihood of survival (OR = 10.39, 95% CI: 4.24–28.07), as well as adherence to haemodynamic GL recommendations (OR = 2.20, 95% CI: 1.01–4.86), while higher epinephrine dose, age, OHCA, and duration of no/low flow were statistically associated with lower odds for ICU survival. Similarly, for survival at ICU discharge with favourable neurological outcome, the shockable rhythm was a positive predictor (OR = 11.19, 95% CI: 4.13–34.65), whereas factors such as age, OHCA and GCS score of 3 at admission were associated with lower odds for this outcome. These results were confirmed also incorporating the year of ICU admission as a random effect instead of the cohort as a fixed effect (Table S9).

**Table 5:** Predictors for survival and favourable neurological outcome at ICU discharge

	Coefficient ± SE	*p*-value	Odds Ratio (95%CI)
**ICU survival**			
Shockable rhythm (ref. No)	2.340 ± 0.479	< 0.0001	10.386 (4.244–28.065)
Adherence– Hemodynamic (ref. No)	0.786 ± 0.398	0.0482	2.195 (1.013–4.863)
Male sex (ref. Female)	0.005 ± 0.469	0.9915	1.005 (0.398–2.537)
Any comorbidity (ref. No)	-0.181 ± 0.615	0.7690	0.835 (0.245–2.778)
Time No-Flow (1 min)	-0.113 ± 0.054	0.0363	0.893 (0.798–0.983)
Time Low-Flow (1 min)	-0.035 ± 0.017	0.0358	0.965 (0.931–0.996)
Out-of-hospital CA (ref. In-hospital CA)	-1.241 ± 0.522	0.0174	0.289 (0.100–0.783)
Age (1 year)	-0.065 ± 0.017	0.0001	0.937 (0.905–0.967)
Epinephrine dose (1 mg)	-0.181 ± 0.092	0.0486	0.834 (0.692–0.995)
**Favourable neurological outcome at ICU discharge**			
Shockable rhythm (ref. No)	2.415 ± 0.539	< 0.0001	11.185 (4.129–34.653)
Cohort (ref. Cohort 1)			
Cohort 2	0.967 ± 0.591	0.1020	2.629 (0.836–8.635)
Cohort 3	0.401 ± 0.623	0.5201	1.493 (0.440–5.159)
Adherence– Hemodynamic (ref. No)	0.581 ± 0.452	0.1990	1.787 (0.742–4.407)
Time No-Flow (1 min)	-0.078 ± 0.058	0.1785	0.925 (0.818–1.016)
GCS = 3 at hospital admission (ref. >3)	-1.326 ± 0.512	0.0096	0.266 (0.094–0.709)
Age (1 year)	-0.048 ± 0.016	0.0023	0.953 (0.923–0.982)
Time Low-Flow (1 min)	-0.035 ± 0.019	0.0667	0.965 (0.927–1.000)
Out-of-hospital CA (ref. in-hospital CA)	-1.571 ± 0.591	0.0079	0.208 (0.062–0.637)
Epinephrine dose (1 mg)	-0.193 ± 0.101	0.0567	0.825 (0.671–1.002)

Abbreviations. CA, cardiac arrest; CI, confidence interval; GCS, Glasgow coma scale; ICU, intensive care unit; SE, standard error

Note. Both models were performed on 185 patients without missing values in predictors

## Discussion

This observational study comprehensively evaluated the impact of evolving guidelines for post-CA care on patients’ outcomes, analysing three distinct consecutive GL periods, i.e. 2010, 2015, and 2021. Overall, an improvement in adherence to GL recommendations was observed across most of the evaluated areas over the study period. Our analysis shows that the implementation of new guideline recommendations follows a progressive trend over time, with increasing adherence across consecutive cohorts. In most macro-areas, this process appears relatively prompt in our institution, reflecting an effective integration into clinical practice. Data showed a consistently high adherence to CAG procedure across all cohorts suggesting that myocardial revascularization has become a well-established practice in post-CA patients. The optimization of haemodynamic has emerged as a key area of improvement across the years with an adherence to GL targets raising from only 32% in 2010 to 77% in 2021, due to a greater attention to all the items composing this area, i.e. MAP, urinary output, and lactate clearance. Indeed, the multivariate analysis confirmed that this specific macro-area of recommendations was an independent predictor for ICU survival and favourable neurological outcome. These findings suggest a clinically relevant benefit associated with the different vasopressor strategies adopted over the years, i.e. increasing use of norepinephrine while reducing dopamine, and highlight the importance of prioritizing MAP and urine output for adequate organ perfusion, targeting the lactate clearance as direct readout of the treatment [15–18].

Differently from haemodynamics, adherence to ventilation recommendations was markedly low, and with a less consistent trend over the years. As highlighted by Battaglini et al., mechanical ventilation should be titrated to prevent both hypoxemia and hyperoxemia while maintaining normal carbon dioxide levels to avoid secondary brain damage, reperfusion injury, and poor outcome [19]. In our cohorts, adherence to TV targets remained relatively high (approximately 70%), while PEEP setting observance increased up to 94% in the last cohort. However, achievement of recommended PaO_2_ and PaCO_2_ targets (10–13 kPa and 4.5–6 kPa, respectively) was notably low across all cohorts, thus affecting the overall ventilation adherence to GLs. Nevertheless, deviations from the above targets were minimal, i.e. PaO_2_ often ranged between 13.3 and 14.7 kPa and PaCO_2_ between 3.3 and 4.5 kPa and 6-6.7 kPa, and probably this was the reason why overall the low adherence to ventilation GLs ultimately did not affect outcomes.

Following emerging evidence, GLs on temperature management in comatose patients resuscitated from CA evolved over the years from mild therapeutic hypothermia (32–34 °C) to TTM (32–36 °C) and TC (< 37.7 °C) [20–22]. Despite these significant modifications, adherence to recommendations significantly increased over time indicating that temperature management has been highly prioritized in post-CA care protocols, although no clear impact on outcome has been proven [23–26]. 

A recent prospective European multicentre cohort study on 337 comatose post-CA patients, demonstrated the effectiveness of the 2021 ERC/ESICM-recommended neuroprognostication algorithm for the prediction of poor neurological outcome [13]. In our analysis, a multimodal strategy for neuroprognostication was adopted in almost all patients in the last 2 cohort periods, reflecting the growing evidence supporting this practice [27]. Notably, a marked increase in the use of quantitative pupillometry was recorded in cohort 3, confirming that this simple but informative bedside tool is now integrated into standard clinical practice in our ICU [28]. In addition, a trend towards more frequent dosage of NSE and use of neuroimaging was observed, confirming increasing attention towards a more comprehensive neurological evaluation [13, 29]. Despite the improved implementation of a multimodal neuroprognostication approach, the rate of WLST, which was approximately 34%, did not show significant increases over the study period.

Over the 14-year study period, survival at ICU discharge increased from 39.5% in cohort 1 to 53.9% in cohort 3, although this trend did not reach statistical significance. Similarly, the proportion of patients with a favourable neurological outcome rose from 30.9 to 42.7%. These proportions are consistent with those reported in recent trials [21, 30]. Regarding outcome predictors, the multivariate analysis identified several important modifiable factors, including pre-hospital aspects such as no/low-flow times and epinephrine use, as well as in-hospital factors such as adherence to guideline recommendations, particularly with respect to hemodynamic management in the ICU. These findings offer potential opportunities for targeted interventions aimed at improving survival and neurological recovery after CA. However, non-modifiable patient characteristics, such as age, gender, and first arrest rhythm, remained strong predictors of outcome, highlighting areas where the potential for intervention is unfortunately limited. These findings are consistent with previous studies [31–33]. 

### Strengths and limitations

The study has several strengths, including its long-term retrospective design covering 14 years and three different GL periods (2010, 2015, 2021), thus providing a comprehensive evaluation of post-CA care evolution. Additionally, the analysis included multiple GL macro-areas along with all individual recommended items, providing a comprehensive multidimensional assessment of GLs implementation. However, some limitations need to be also acknowledged. First, despite the characteristics of the study population, including demographic, anamnestic and CA/CPR data, appearing consistent with other European realities, the single-centre design might limit the generalizability of the results [1]. Second, the retrospective design of the study and the reliance on medical records to identify the study population may have introduced potential selection bias. However, to overcome this issue, two independent records reviewers were enrolled with the adjunct of a senior supervisor. Third, we would like to highlight that our adherence measure is based on achieved values rather than specific treatment, which may not fully capture clinician intent. Moreover, the definition of adherence to MAP targets was based on measured values, without distinguishing whether deviations reflected inadequate compliance with treatment protocols or the clinical impossibility of achieving targets despite appropriate interventions. This may lead to an underestimation of adherence in patients with refractory hypotension and further analyses are needed. Moreover, the presence of competing risks, such as early death, may have influenced the assessment of adherence by reducing the available time to implement certain recommended interventions. However, we adopted several specific strategies to minimize this potential bias, including the exclusion of patients who died within 24 h, the removal of terminal days from the adherence analysis, and the use of a conservative adherence definition to enhance the robustness of our evaluation. In this context, the definition of overall compliance as adherence being present on more than 50% of the observation days is arbitrary and may be considered subjective. This threshold was chosen to also reflect a majority trend, but it may not capture clinically meaningful adherence patterns and should be interpreted with caution. Finally, the exclusion of the SOFA score in the analysis, due to a high proportion of missing data (Table S1), may have limited our ability to fully assess patients’ severity at admission. Age, CCI, comorbidities, and GCS were used as proxies to address this gap. Future studies could benefit from a prospective design and more complete data collection to mitigate these limitations.

## Conclusions

This retrospective 14-year study, covering three consecutive guidelines period, i.e. 2010, 2015, and 2021, demonstrates significant improvements in adherence to key post-CA care guideline recommendations, particularly in haemodynamic optimization, temperature control, and neuroprognostication. Although these findings suggest a beneficial impact of guidelines implementation, the increased adherence to recommendations over time did not lead to statistically significant improvements in survival and neurological outcome.

Future larger multicentre studies are needed to validate these results and further refine post-CA care guidelines.

## Electronic supplementary material

Below is the link to the electronic supplementary material.

Supplementary Material 1

Supplementary Material 2

## Data Availability

The datasets used and/or analysed during the current study are available from the corresponding author on reasonable request. The study report adheres to the Strengthening the Reporting of Observational Studies in Epidemiology (STROBE) Statement (Supplementary Table S10) [34].

## Abbreviations

AED: Antiepileptic drugs
ANOVA: Analysis of variance
CA: Cardiac arrest
CAG: Coronary angiography
CI: Confidence interval
CCI: Charlson comorbidity index
CPC: Cerebral performance category
CPR: Cardiopulmonary resuscitation
CT: Computed tomography
DVTP: Deep venous thrombosis prophylaxis
ECMO V-A: Extracorporeal membrane oxygenation veno-arterial
EEG: Electroencephalogram
ERC: European resuscitation council
ESICM: European society of intensive care medicine
FiO_2_: Fraction of inspired oxygen
GCS: Glasgow coma scale
GLs: Guidelines
ICU: Intensive care unit
IQR: Interquartile range (1st quartile– 3rd quartile)
LASSO: Least absolute shrinkage and selection operator
LOS: Length of stay
MAP: Mean arterial pressure
MRI: Magnetic resonance imaging
NSE: Neuron serum enolase
OHCA: Out of hospital cardiac arrest
OR: Odds ratio
PaO_2_: Arterial oxygen pressure
PaCO_2_: Arterial carbon dioxide pressure
PCI: Percutaneous coronary intervention
PEEP: Positive end expiratory pressure
ROSC: Return of spontaneous circulation
SaO_2_: Arterial oxygen saturation
SD: Standard deviation
SOFA: Sequential organ failure assessment
SSEP: Somatosensory evoked potential
STROBE: Strengthening the reporting of observational studies in epidemiology
SUP: Stress ulcer prophylaxis
TC: Temperature control
TTM: Targeted temperature management
TV: Tidal volume
WLST: Withdrawal of life-sustaining therapy
