# Supplementary material for: Adherence to post-cardiac arrest care guidelines and impact on survival and neurological outcome
Source: Ann Intensive Care. 2025 Jul 2;15:88. doi: 10.1186/s13613-025-01508-1 (PMC12222584; doi:10.1186/s13613-025-01508-1)
Supplement: Supplementary file 1 — Supplementary Material 1 [file 13613_2025_1508_MOESM1_ESM.docx]

**Supplementary appendix**

**Adherence to Post-Cardiac Arrest Care Guidelines and Impact on**

**Survival and Neurological Outcome**

**AUTHORS**

Giulia Merigo, Fabiana Madotto, Aurora Magliocca, Gaetano Florio, Alessandra Rosati, Valentina Castagna, Marco Pagliano, Alberto Zanella, Mauro Panigada, Giacomo Grasselli, Giuseppe Ristagno

**Methods**

**Adherence definition**

Adherence was defined as clinical values staying within the recommended range, or deviating only once (i.e., not more than one consecutive out-of-range measurement). Non-adherence was defined when two or more consecutive values (taken at least six hours apart) fell outside the recommended range. This evaluation was performed daily for each item of guidelines, until four days after ICU admission. An item was considered overall compliant if adherence was observed on more than half of the monitored days.

A macro-area was considered adherent to guidelines if all its items met the compliance criteria above. The only exception was the multimodal neuroprognostication macro-area, where adherence required at least two neurological items to be completed, in accordance with GL recommendations.

**Statistical analysis**

Considering three cohorts of post-CA patients admitted to the ICU, the study aimed to test whether there was a positive linear trend in the proportion of survivors at ICU discharge (H0: p1 = p2 = p3 vs H1: p1 < p2 < p3), with a Type I error probability of 5% (alpha) and a power of 80% (1-beta). Assuming survival rates of 25% (p1), 35% (p2), and 45% (p3) in the three cohorts, the study planned to enrol at least 70 patients in each cohort (for a total of 210 patients) to test the hypothesis using the Cochran–Armitage trend test.

According to data distribution, continuous variables were reported as mean and standard deviation (SD) or as median and interquartile range (first and third quartiles) (IQR). Categorical variables are expressed as counts and percentages. No imputation procedures were used for missing values. Number of patients with missing data for each variable of interest was reported in **Supplementary Tables S1.** For categorical variables, comparisons among the 3 cohorts were performed using the Chi-square test or Fisher’s exact test, as appropriate. The Cochran-Armitage trend test was used to assess linear trends in the proportions of categorical variables across cohorts. For continuous variables, one-way Analysis of Variance (ANOVA) was used when the variable was normally distributed, as assessed by the Shapiro-Wilk test. For continuous variables not Normally distributed, the Kruskal-Wallis test was applied, and if necessary, the variables were transformed using the Box-Cox transformation to achieve normality. For post-hoc pairwise comparisons between cohorts, Dunnett’s test was used, with the cohort 1 as the reference group.

To investigate the factors influencing survival, the following variables were considered as potential predictors for ICU survival: cohorts (3 levels), adherence (yes, no) to the macro-areas of post-CA care GL (haemodynamic, ventilation, TTM and ICU management), sex (male, female), out-of-hospital CA (OHCA) (yes, no), cardiac aetiology (yes, no), shockable rhythm (yes, no), lay bystander CPR (yes, no), mechanical chest compression (yes, no), epinephrine dose (mg), comorbidities (yes, no), drug treatment (yes, no), age (years), no-flow time (minutes), low-flow time (minutes), Sequential Organ Failure Assessment (SOFA) score, Charlson Comorbidity Index (CCI) and GCS at hospital admission. Adherence to the coronary angiography, neuroprognostication, and seizure control macro-areas was not included as predictors because these interventions were not uniformly indicated across all patient groups or guideline periods. This decision aimed to avoid confounding by indication and ensure comparability across the cohorts. The effect of adherence to each single item within the macro-areas was also investigated. Variables with >20% of missing data or a prevalence <10% or >90% were not considered as possible predictors.

Separate univariate logistic regression models with survival at ICU discharge (survived, non-survived) and survival at ICU discharge with favourable neurological outcome (alive with CPC score 1 or 2, or death/alive with CPC score >2 at ICU discharge) as the dependent variables, were performed. All continuous variables were standardized in order to compare predictors with different units of measurement (effect sizes on the same scale). Potential confounders, which could influence the relationship between predictors and the outcomes, were considered when selecting variables for the univariate analysis. Assumptions for the logistic regression models (linearity, independence, multicollinearity) were assessed. Following the univariate analysis, we applied the Least Absolute Shrinkage and Selection Operator (LASSO) using logistic regression to identify the most significant predictors for inclusion in a multivariable logistic regression model. LASSO addresses issues of multicollinearity and overfitting by applying a penalty to less relevant variables, resulting in a more parsimonious and generalizable model. Model selection was performed through cross-validation, and the dataset was randomly split into 80% for training and 20% for testing to assess model accuracy. In detail, the final LASSO model, trained on the training set, was applied to the test dataset to generate predicted probabilities. Predictions were classified as 1 (survived) if the predicted probability was greater than 50%, and as 0 otherwise. Accuracy was determined by calculating the proportion of correct predictions compared to the true outcome from the test set. Variables selected by LASSO were subsequently entered into unpenalized multivariable logistic regression models, using the non-standardized continuous variables, to estimate the odds ratios (OR) of the identified predictors, allowing for an unbiased estimation of the relationship between the predictors and the outcomes (magnitude and direction), without the influence of regularization. Moreover, to account for temporal changes and improvements in post-cardiac arrest care, we performed multivariate mixed-effects models with the year of ICU admission included as a random effect.

To estimate the temporal trend in adherence, a generalized linear model with a binomial family was used for each area and item of guidelines recommendations. The response variable consisted of the yearly proportion of adherents, while the predictor was a cubic spline of the year of admission (with 3 degrees of freedom) to capture the trend over time.

All statistical tests were two-sided with a significance α-level of 0.05. Analyses were performed using R 4.3.2 (R Foundation for Statistical Computing, Vienna, Austria) and SAS 9.4 (SAS Institute, Cary, North Carolina, USA).

**Results**

**Figure S1.** Annual trend in adherence to current and previous post-CA GL recommendations, by single item.

*For each item of guideline (GL) recommendations, the figure shows the annual percentage of patients adherent to the GL in force at the time of cardiac arrest (red dots) and those who remained adherent to the previous GL (blue dots). When the recommendation remains unchanged or similar across both GLs, the two dots overlap, resulting in a purple-grey appearance. Grey vertical bands indicate the publication of a new GL. In 2021, more than two dots may appear because two GL versions were in effect, with the most recent published in March 2021.*


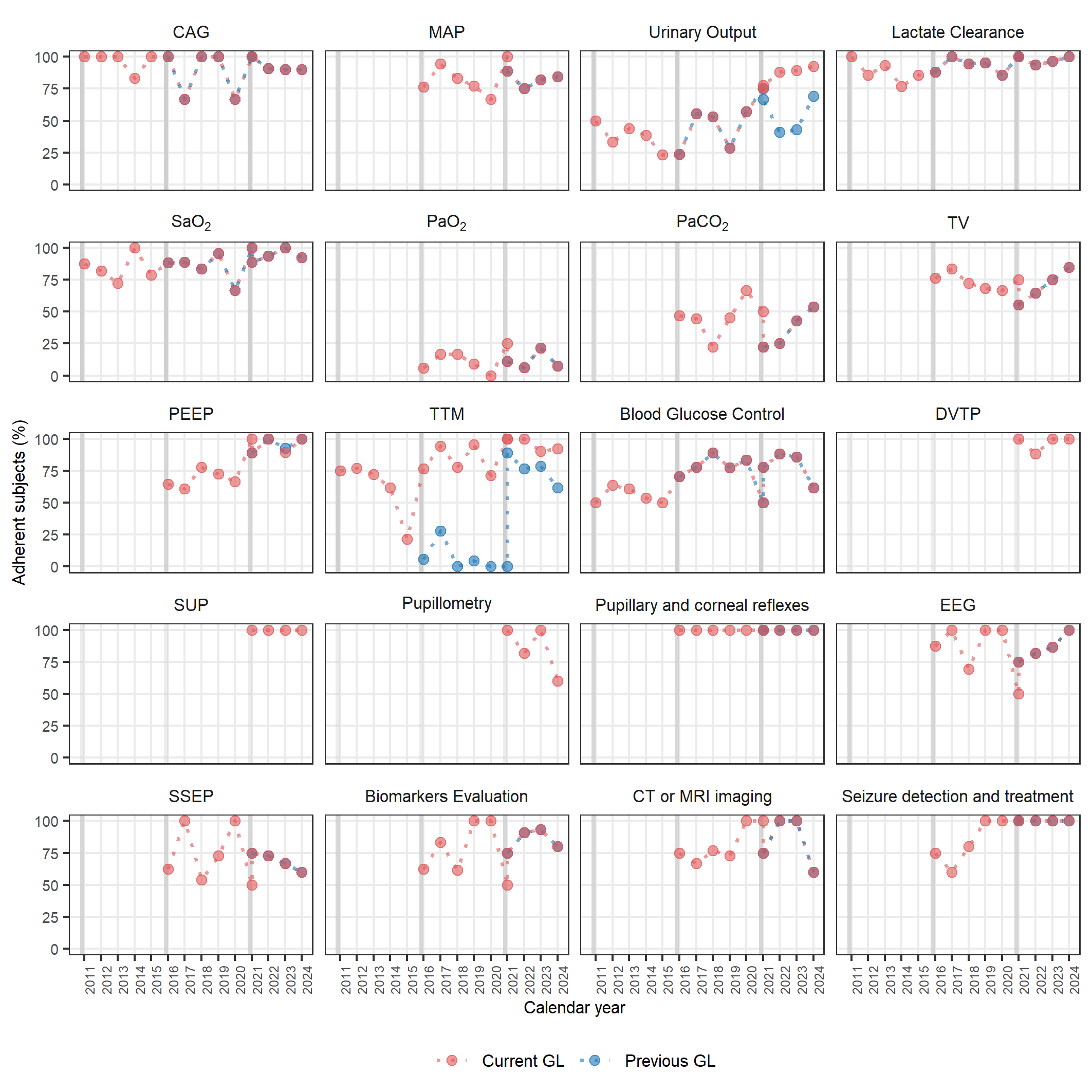


**Table S1.** Number of patients with missing data for each variable of interest.

|  | **All patient**  **(n = 275)** |
| --- | --- |
| **Demographic characteristics, n (%)** |  |
| Sex | 0 (0.00) |
| Age (years) | 0 (0.00) |
| CCI (score) | 22 (8.00) |
| **CA and CPR characteristics, n (%)** |  |
| OHCA | 0 (0.00) |
| Pathogenesis | 0 (0.00) |
| Rhythm | 1 (0.36) |
| Witnessed cardiac arrest | 0 (0.00) |
| Lay Bystander CPR | 0 (0.00) |
| No flow (min) | 0 (0.00) |
| Low flow (min) | 7 (2.55) |
| Mechanical CC | 0 (0.00) |
| Epinephrine dose (mg) | 9 (3.27) |
| Amiodarone use | 1 (0.36) |
| Defibrillation attempts | 4 (1.45) |
| Any comorbidities | 0 (0.0) |
| Active drug treatment | 0 (0.00) |
| SOFA score | 129 (46.91) |
| **Treatments and procedures, n (%)** |  |
| Coronary angiography | 0 (0.00) |
| PCI | 1 (0.36) |
| ECMO V-A | 0 (0.00) |
| ECMO V-V | 0 (0.00) |
| IABP | 0 (0.00) |
| CRRT | 0 (0.00) |
| Antiepileptic drugs administered | 0 (0.00) |
| Inotropic/vasopressor drugs administered | 3 (1.09) |
| Epinephrine IV use | 3 (1.09) |
| Norepinephrine IV use | 3 (1.09) |
| Dobutamine IV use | 3 (1.09) |
| Dopamine IV use | 3 (1.09) |
| **Multimodal neuroprognostication, n (%)** |  |
| TTM | 0 (0.00) |
| Pupillometry (NPI) | 0 (0.00) |
| Pupillary and corneal reflexes | 0 (0.00) |
| EEG | 0 (0.00) |
| SSEP | 1 (0.36) |
| Brain MRI | 0 (0.00) |
| Brain CT | 0 (0.00) |
| NSE dosage | 0 (0.00) |
| NSE peak value (mcg/L) | 0 (0.00) |
| Early myoclonus status | 0 (0.00) |
| **Primary endpoints, n (%)** |  |
| ROSC | 18 (6.55) |
| Alive at ICU discharge | 0 (0.00) |
| **Secondary endpoints, n (%)** |  |
| Favourable neurological outcome at ICU discharge | 0 (0.00) |
| 1-month survival | 3 (1.09) |
| 1-month survival with favourable neurological outcome | 3 (1.09) |
| 6-month survival | 23 (8.36) |
| 6-month survival with favourable neurological outcome | 23 (8.36) |
| WLST | 0 (0.00) |
| **Other clinical outcomes, n (%)** |  |
| Length of ICU stay (days) | 0 (0.00) |
| Length of mechanical ventilation (days) | 5 (1.82) |
| Length of hospital stay (days) | 1 (0.36) |
| **Adherence to GLs, n (%*)** |  |
| **Coronary angiography^†^** | 0 (0.00) |
| Overall adherence^†^ | 0 (0.00) |
| **Haemodynamic** |  |
| Overall adherence | 21 (9.50) |
| MAP^‡^ | 2 (1.31) |
| Urinary output | 20 (9.05) |
| Lactate | 6 (2.71) |
| **Ventilation** |  |
| Overall adherence | 6 (2.71) |
| SaO_2_ | 6 (2.71) |
| TV^‡^ | 1 (0.65) |
| PEEP^‡^ | 1 (0.65) |
| PaO_2_^‡^ | 2 (1.31) |
| PaCO_2_^‡^ | 2 (1.31) |
| **Temperature management** |  |
| Overall adherence | 15 (6.79) |
| **General ICU management** |  |
| Overall adherence | 5 (2.26) |
| Glycaemia | 5 (2.26) |
| DVTP^§^ | 0 (0.00) |
| SUP^§^ | 0 (0.00) |
| **Multimodal neuroprognostication** |  |
| Overall adherence^#^ | 0 (0.00) |
| Pupillometry^^^ | 0 (0.00) |
| Pupillary and corneal reflexes^#^ | 0 (0.00) |
| SSEP^#^ | 0 (0.00) |
| EEG^#^ | 0 (0.00) |
| NSE^#^ | 0 (0.00) |
| CT or MRI imaging^#^ | 0 (0.00) |
| **Seizures treatment** |  |
| Overall adherence^£^ | 0 (0.00) |

*Abbreviations. CA, cardiac arrest; CC, chest compression; CCI, Charlson’s comorbidity index; CPR, cardiopulmonary resuscitation; CRRT, continuous renal replacement therapy; CT, computed tomography; DVTP, deep venous thrombosis prophylaxis; ECMO, extracorporeal membrane oxygenation; EEG, electroencephalography; GL, guideline; IABP, intra-aortic balloon pump; ICU, intensive care unit; MAP, mean arterial pressure; MRI, magnetic resonance imaging; NPI, neurological prognostic index; NSE, neuron serum enolase; OHCA, out-of-hospital cardiac arrest; PaCO_2_, arterial carbon dioxide pressure; PaO_2_, arterial oxygen pressure; PCI, percutaneous coronary intervention; PEEP, positive end expiratory pressure; SaO_2_, arterial oxygen saturation; SSEP, somatosensory evoked potentials; SOFA, sequential organ failure assessment; STEMI, ST-elevation myocardial infarction; SUP, stress ulcer prophylaxis; TTM, targeted temperature management; TTM/TC, targeted temperature management/controlled hypothermia; TV, tidal volume; V-A, venous-arterial; V-V, venous-venous.*

** The percentage was calculated based on 221 patients who were comatose for at least 24 hours, in accordance with GL recommendations.*

*†The percentage was calculated based on 78 patients with STEMI who were comatose for at least 24 hours, in accordance with GL recommendations.*

*^‡^ The percentage was calculated based on 153 comatose patients (for at least 24 hours) belonging to cohorts 2 and 3.*

*^§^ The percentage was calculated based on 67 comatose patients (for at least 24 hours) belonging to cohort 3.*

*^#^ The percentage was calculated based on 78 patients who were comatose for at least 72 hours (in accordance with GL recommendations) belonging to cohort 2 and 3.*

*^^^ The percentage was calculated based on 35 patients who were comatose for at least 72 hours (in accordance with GL recommendations) belonging to cohort 3.*

*^£^ The percentage was calculated based on 42 patients with seizure who were comatose for at least 72 hours (in accordance with GL recommendations) belonging to cohort 3.*

**Table S2.** Cardiac arrest characteristics in the study population, stratified by cohort.

|  | **All patients**  (n=275) | **Cohort 1**  **GL 2010**  ***(01/2011–12/2015)***  (n=81) | **Cohort 2**  **GL 2015**  ***(01/2016–03/2021)***  (n=105) | **Cohort 3**  **GL 2021**  ***(04/2021–09/2024)***  (n=89) | **p-value** |  |  |
| --- | --- | --- | --- | --- | --- | --- | --- |
| Cardiac aetiology, n (%) | 163 (59.49) | 50 (62.50) | 51 (48.57) | 62 (69.66) | 0.0095 |  |  |
| AMI and MINOCA^§^ | 102 (62.58) | 25 (50.00) | 30 (58.82) | 47 (75.81) ^†^ | 0.0156 |  |  |
| **AMI classification°, n (%)** | | | | | | |  |
| STEMI | 86 (85.15) | 22 (88.00) | 24 (80.00) | 40 (86.96) | 0.7801 |  |  |
| NSTEMI | 7 (6.93) | 3 (12.00) | 4 (13.33) | 0 (0.00) | 0.0195 |  |  |
| MINOCA | 8 (7.92) | 0 (0.00) | 2 (6.67) | 6 (13.04) | 0.1723 |  |  |
| **STEMI location, n (%) *** | | | | | 0.2394 |  |  |
| Anterior | 35 (40.70) | 11 (50.00) | 9 (37.50) | 15 (37.50) | - |  |  |
| Inferior | 18 (20.93) | 6 (27.27) | 2 (8.33) | 10 (25.00) | - |  |  |
| Lateral | 4 (4.65) | 0 (0.00) | 2 (8.33) | 2 (5.00) | - |  |  |
| Posterior | 3 (3.49) | 0 (0.00) | 0 (0.00) | 3 (7.50) | - |  |  |
| Missing location | 26 (30.23) | 5 (22.73) | 11 (45.83) | 10 (25.00) | - |  |  |
| **First arresting rhythm, n (%)** | | | | | | | |
| VF ^§^ | 113 (41.24) | 30 (37.04) | 35 (33.65) | 48 (53.93) ^†^ | 0.0112 |  |  |
| VT | 9 (3.28) | 1 (1.23) | 5 (4.81) | 3 (3.37) | 0.4371 |  |  |
| Asystole ^§^ | 83 (30.29) | 29 (35.80) | 38 (36.54) | 16 (17.98) ^†^ | 0.0087 |  |  |
| PEA | 69 (25.18) | 21 (25.93) | 26 (25.00) | 22 (24.72) | 0.9823 |  |  |
| **Clinical severity upon ICU admission, median [IQR]** | | | | | | |  |
| SOFA components |  |  |  |  |  |  |  |
| Neurologic | 4.00 [4.00-4.00] | 4.00 [4.00-4.00] | 4.00 [4.00-4.00] ^†^ | 4.00 [4.00-4.00] | 0.0291 |  |  |
| Cardiovascular | 4.00 [1.00-4.00] | 3.00 [0.00-4.00] | 4.00 [2.00-4.00] ^†^ | 4.00 [3.00-4.00] ^†^ | 0.0052 |  |  |
| Respiratory | 2.00 [1.00-3.00] | 2.00 [2.00-3.00] | 2.00 [0.00-3.00] ^†^ | 1.50 [0.00-3.00] ^†^ | 0.0014 |  |  |
| Coagulation | 0.00 [0.00-0.00] | 0.00 [0.00-0.00] | 0.00 [0.00-0.00] | 0.00 [0.00-0.00] | 0.2959 |  |  |
| Liver | 0.00 [0.00-0.00] | 0.00 [0.00-0.00] | 0.00 [0.00-0.00] | 0.00 [0.00-0.00] | 0.9654 |  |  |
| Renal function | 1.00 [0.00-1.00] | 0.00 [0.00-1.00] | 1.00 [0.00-1.00] | 0.00 [0.00-1.00] | 0.4563 |  |  |
| GCS Hospital admission | 3.00 [3.00-3.00] | 3.00 [3.00-5.00] | 3.00 [3.00-3.00] ^†^ | 3.00 [3.00-4.00] | 0.0141 |  |  |

*Abbreviations. AMI, acute myocardial infarction; GCS, Glasgow coma scale; GL, guideline; ICU, intensive care unit; IQR, interquartile range [1^st^ quartile-3^rd^ quartile]; MINOCA, myocardial infarction with non-obstructive coronary arteries; PEA, pulseless electrical activity; SOFA, sequential organ failure assessment; VF, ventricular fibrillation; VT, ventricular tachycardia*

*† p <0.05, comparison vs “Cohort 1” (adjusted for multiple comparison, Dunnett’s test). § p <0.05, Cochran Armitage test for trend.*

*° Percentages were calculated using the number of patients with AMI/MINOCA as denominator (n=102).*

** Percentages were calculated using the number of patients with STEMI as denominator (n=86).*

**Table S3.** Prevalence of comorbidities at ICU admission in the study population, stratified by cohort.

|  | **All patients**  (n=275) | **Cohort 1**  **GL 2010**  ***(01/2011–12/2015)***  (n=81) | **Cohort 2**  **GL 2015**  ***(01/2016–03/2021)***  (n=105) | **Cohort 3**  **GL 2021**  ***(04/2021–09/2024)***  (n=89) | **p-value** |  |
| --- | --- | --- | --- | --- | --- | --- |
| **Comorbidities at ICU admission, n (%)** | | | | | | |
| Potus | 42 (15.27) | 12 (14.81) | 14 (13.33) | 16 (17.98) | 0.6632 |  |
| Former or active smoker | 103 (37.45) | 26 (32.10) | 37 (35.24) | 40 (44.94) | 0.1880 |  |
| Obesity | 32 (11.64) | 9 (11.11) | 10 (9.52) | 13 (14.61) | 0.5376 |  |
| Family history of CVD ^§^ | 26 (9.45) | 1 (1.23) | 11 (10.48) ^†^ | 14 (15.73) ^†^ | 0.0050 |  |
| Syncopal episode | 39 (14.18) | 10 (12.35) | 19 (18.10) | 10 (11.24) | 0.3362 |  |
| Depressive disorders | 42 (15.27) | 8 (9.88) | 21 (20.00) | 13 (14.61) | 0.1599 |  |
| Cerebrovascular disease ^§^ | 17 (6.18) | 7 (8.64) | 9 (8.57) | 1 (1.12) | 0.0549 |  |
| Diabetes | 54 (19.64) | 18 (22.22) | 23 (21.90) | 13 (14.61) | 0.3478 |  |
| Cerebral haemorrhage | 3 (1.09) | 1 (1.23) | 0 (0.00) | 2 (2.25) | 0.2893 |  |
| Peripheral neuropathy | 6 (2.18) | 3 (3.70) | 3 (2.86) | 0 (0.00) | 0.2202 |  |
| Previous ischemic cardiopathy | 63 (22.91) | 18 (22.22) | 24 (22.86) | 21 (23.60) | 0.9775 |  |
| Previous PTCA | 30 (13.57) | 7 (10.29) | 11 (12.79) | 12 (17.91) | 0.4184 |  |
| TIA | 18 (6.55) | 8 (9.88) | 6 (5.71) | 4 (4.49) | 0.3329 |  |
| Malignant tumor | 10 (3.64) | 2 (2.47) | 6 (5.71) | 2 (2.25) | 0.4721 |  |
| Hypertension | 126 (45.82) | 41 (50.62) | 51 (48.57) | 34 (38.20) | 0.2068 |  |
| Dyslipidaemia | 68 (24.73) | 18 (22.22) | 29 (27.62) | 21 (23.60) | 0.6683 |  |
| Peptic ulcer disease ^§^ | 20 (7.27) | 1 (1.23) | 7 (6.67) | 12 (13.40) ^†^ | 0.0085 |  |
| Peripheral vascular disease ^§^ | 10 (3.64) | 7 (8.64) | 1 (0.95) ^†^ | 2 (2.25) | 0.0203 |  |
| Mitral valve insufficiency ^§^ | 11 (4.00) | 7 (8.64) | 4 (3.81) | 0 (0.00) ^†^ | 0.0120 |  |
| Atrial fibrillation | 29 (10.55) | 14 (17.28) | 7 (6.67) | 8 (8.99) | 0.0550 |  |
| Pneumothorax ^§^ | 4 (1.45) | 3 (3.70) | 1 (0.95) | 0 (0.00) | 0.1091 |  |
| COPD | 36 (13.09) | 15 (18.52) | 13 (12.38) | 8 (8.99) | 0.1773 |  |
| OSAS | 8 (2.91) | 1 (1.23) | 4 (3.81) | 3 (3.37) | 0.6651 |  |
| Liver disease | 15 (5.45) | 4 (4.94) | 7 (6.67) | 4 (4.49) | 0.7978 |  |
| Renal disease | 25 (9.09) | 9 (11.11) | 10 (9.52) | 6 (6.74) | 0.6010 |  |
| Transplant | 8 (2.91) | 4 (4.94) | 1 (0.95) | 3 (3.37) | 0.2495 |  |

*Abbreviations. COPD, chronic obstructive pulmonary disease; CVD, cardiovascular disease; GL, guideline; ICU, intensive care unit; OSAS, obstructive sleep apnoea syndrome; PTCA, percutaneous transluminal coronary angioplasty; TIA, transient ischemic attack*

*† p <0.05, comparison vs “Cohort 1” (adjusted for multiple comparison, Dunnett’s test). § p <0.05, Cochran Armitage test for trend.*

**Table S4.** Active drug treatments at ICU admission in the study population, stratified by cohort.

|  | **All patients**  (n=275) | **Cohort 1**  **GL 2010**  ***(01/2011–12/2015)***  (n=81) | **Cohort 2**  **GL 2015**  ***(01/2016–03/2021)***  (n=105) | **Cohort 3**  **GL 2021**  ***(04/2021–09/2024)***  (n=89) | **p-value** |  |
| --- | --- | --- | --- | --- | --- | --- |
| **Active drug treatment, n (%)** | | | | | | |
| Cardiac therapy | 20 (7.27) | 5 (6.17) | 8 (7.62) | 7 (7.87) | 0.9002 |  |
| Antithrombotic drugs | 88 (32.00) | 22 (27.16) | 36 (34.29) | 30 (33.71) | 0.5371 |  |
| Diuretics | 40 (14.55) | 14 (17.28) | 17 (16.19) | 9 (10.11) | 0.3457 |  |
| β-blockers | 58 (21.09) | 16 (19.75) | 23 (21.90) | 19 (21.35) | 0.9359 |  |
| Calcium channel blockers | 12 (4.36) | 2 (2.47) | 7 (6.67) | 3 (3.37) | 0.3694 |  |
| Renin-angiotensin system antagonists | 58 (21.09) | 13 (16.05) | 24 (22.86) | 21 (23.60) | 0.4129 |  |
| Lipid-modifying agents | 49 (17.82) | 10 (12.35) | 18 (17.14) | 21 (23.60) | 0.1558 |  |
| Drugs for obstructive airway diseases | 7 (2.55) | 1 (1.23) | 5 (4.76) | 1 (1.12) | 0.2682 |  |
| Peptic ulcer and gastro-oesophageal reflux disease | 41 (14.91) | 10 (12.35) | 16 (15.24) | 15 (16.85) | 0.7069 |  |
| Antidiabetic | 25 (9.09) | 6 (7.41) | 10 (9.52) | 9 (10.11) | 0.8130 |  |
| Glucocorticoids | 6 (2.18) | 1 (1.23) | 4 (3.81) | 1 (1.12) | 0.3876 |  |
| Antineoplastic and immunomodulating | 4 (1.45) | 2 (2.47) | 1 (0.95) | 1 (1.12) | 0.6883 |  |
| Anti-seizure medications | 10 (3.64) | 0 (0.00) | 6 (5.71) | 4 (4.49) | 0.0738 |  |
| Psycholeptics and psychoanaleptics drugs | 35 (12.73) | 7 (8.64) | 16 (15.24) | 12 (13.48) | 0.3948 |  |
| Other drugs | 10 (3.64) | 1 (1.23) | 6 (5.71) | 3 (3.37) | 0.2824 |  |
| **Concurrent drug treatments, n (%)** | | | | | 0.1393 |  |
| 0 | 115 (41.82) | 40 (49.38) | 35 (33.33) | 40 (44.94) | - |  |
| 1-2 | 76 (27.64) | 23 (28.40) | 33 (31.43) | 20 (22.47) | - |  |
| 3-4 | 50 (18.18) | 12 (14.81) | 24 (22.86) | 14 (15.73) | - |  |
| >4 | 34 (12.36) | 6 (7.41) | 13 (12.38) | 15 (16.85) | - |  |

*Abbreviations. GL, guideline.*

**Table S5.** Post-cardiac arrest management in the comatose patients (≥24 h), stratified by cohort

|  | **All patients**  (n=221) | **Cohort 1**  **GL 2010**  ***(01/2011–12/2015)***  (n=68) | **Cohort 2**  **GL 2015**  ***(01/2016–03/2021)***  (n=86) | **Cohort 3**  **GL 2021**  ***(04/2021–09/2024)***  (n=67) | **p-value** |  |
| --- | --- | --- | --- | --- | --- | --- |
| **Treatments and procedures, n (%)** | | | | | | |
| Coronary angiography | 92 (41.63) | 25 (36.76) | 28 (32.56) | 39 (58.21) ^†^ | 0.0044 |  |
| PCI | 81 (36.82) | 23 (34.33) | 25 (29.07) | 33 (49.25) | 0.0347 |  |
| ECMO V-A | 28 (12.67) | 5 (7.35) | 11 (12.79) | 12 (17.91) | 0.1825 |  |
| ECMO V-V | 2 (0.90) | 1 (1.47) | 1 (1.16) | 0 (0.00) | 1.0000 |  |
| IABP | 34 (15.38) | 12 (17.65) | 5 (5.81) ^†^ | 17 (25.37) | 0.0033 |  |
| CRRT | 24 (10.86) | 9 (13.24) | 10 (11.63) | 5 (7.46) | 0.5359 |  |
| Antiepileptic drugs administered | 58 (26.24) | 20 (29.41) | 22 (25.58) | 16 (23.88) | 0.7538 |  |
| Inotropic/vasopressor drugs administered | 162 (73.30) | 45 (66.18) | 65 (75.58) | 52 (77.61) | 0.2687 |  |
| Epinephrine IV use | 97 (43.89) | 27 (39.71) | 36 (41.86) | 34 (50.75) | 0.3856 |  |
| Norepinephrine IV use **^§^** | 95 (42.99) | 13 (19.12) | 44 (51.16) ^†^ | 38 (56.72) ^†^ | <.0001 |  |
| Dobutamine IV use **^§^** | 18 (8.14) | 9 (13.24) | 7 (8.14) | 2 (2.99) | 0.0935 |  |
| Dopamine IV use **^§^** | 23 (10.41) | 15 (22.09) | 6 (6.98) ^†^ | 2 (2.99) ^†^ | 0.0006 |  |
| **Multimodal neuroprognostication, n (%)** | | | | | | |
| TTM/TC **§** | 171 (77.38) | 44 (64.71) | 74 (86.05) ^†^ | 53 (79.10) | 0.0066 |  |
| Pupillometry (NPI) **^§^** | 69 (31.22) | 0 (0.00) | 13 (15.12) ^†^ | 56 (83.58) ^†^ | <.0001 |  |
| Pupillary and corneal reflexes **^§^** | 189 (85.52) | 52 (76.47) | 76 (88.37) | 61 (91.04) | 0.0348 |  |
| EEG **^§^** | 146 (66.06) | 37 (54.41) | 62 (72.09) ^†^ | 47 (70.15) | 0.0495 |  |
| SSEP **^§^** | 71 (32.27) | 5 (7.35) | 41 (48.24) ^†^ | 25 (37.31) ^†^ | <.0001 |  |
| Brain MRI **^§^** | 11 (4.98) | 1 (1.47) | 3 (3.49) | 7 (10.45) ^†^ | 0.0550 |  |
| Brain CT | 154 (69.68) | 38 (55.88) | 55 (63.95) | 61 (91.04) ^†^ | 0.0001 |  |
| Patients with NSE measured **^§^** | 130 (57.47) | 25 (36.76) | 57 (66.28) ^†^ | 45 (67.16) ^†^ | 0.0002 |  |
| NSE peak value (mcg/L), median [IQR] | 31.60 [17.20-96.10] | 31.60 [17.20-80.50] | 30.10 [14.40-132.40] | 32.60 [19.10-72.00] | 0.9502 |  |
| Patients with early myoclonus status | 54 (24.43) | 17 (25.00) | 24 (27.91) | 13 (19.40) | 0.4742 |  |

*Abbreviations. CRRT, continuous renal replacement therapy; CT, computed tomography; ECMO, extracorporeal membrane oxygenation; EEG, electroencephalography; GL, guideline; IABP, intra-aortic balloon pump; IQR, interquartile range [1^st^ quartile – 3^rd^ quartile]; MRI, magnetic resonance imaging; NPI, neurological prognostic index; NSE, neuron serum enolase; PCI, percutaneous coronary intervention; SSEP, somatosensory evoked potentials; TTM/TC, targeted temperature management/controlled hypothermia; V-A, venous-arterial; V-V, venous-venous.*

*† p <0.05, comparison vs “Cohort 1” (adjusted for multiple comparison, Dunnett’s test). § p <0.05, Cochran Armitage test for trend.*

**Table S6.** Clinical outcomes in the comatose patients (≥24 h), stratified by cohort

|  | **All patients**  (n=221) | **Cohort 1**  **GL 2010**  ***(01/2011–12/2015)***  (n=68) | **Cohort 2**  **GL 2015**  ***(01/2016–03/2021)***  (n=86) | **Cohort 3**  **GL 2021**  ***(04/2021–09/2024)***  (n=67) | **p-value** |
| --- | --- | --- | --- | --- | --- |
| **Primary endpoints, n (%)** | | | | | |
| Alive at ICU discharge | 106 (47.96) | 28 (41.18) | 40 (46.51) | 38 (56.72) | 0.1841 |
| **Secondary endpoints, n (%)** | | | | | |
| Patients with favourable neurological outcome at ICU discharge | 88 (39.82) | 22 (32.35) | 36 (41.86) | 30 (44.78) | 0.2319 |
| 1-month survival **§** | 95 (43.58) | 23 (33.82) | 37 (43.02) | 35 (54.69) | 0.0535 |
| 1-month survival with favourable neurological outcome **§** | 79 (36.24) | 18 (26.47) | 33 (38.37) | 28 (43.75) | 0.1033 |
| 6-month survival | 74 (37.00) | 21 (33.33) | 27 (34.62) | 26 (44.07) | 0.4029 |
| 6-month survival with favourable neurological outcome | 65 (32.20) | 18 (28.57) | 26 (33.33) | 21 (35.59) | 0.6958 |
| WLST | 81 (36.65) | 20 (29.41) | 36 (41.86) | 25 (37.31) | 0.2791 |
| **Other clinical outcomes, median [IQR]** | | | | | |
| Length of ICU stay (days) | 5.00 [4.00-8.00] | 5.00 [4.00-7.00] | 5.00 [4.00-8.00] | 6.00 [3.00-9.00] | 0.4260 |
| Length of mechanical ventilation (days) | 4.00 [3.00-7.00] | 4.00 [3.00-6.00] | 4.00 [3.00-6.00] | 4.00 [2.00-8.00] | 0.8524 |
| Length of hospital stay (days) | 10.00 [5.00-19.00] | 8.00 [4.00-20.00] | 10.00 [5.00-18.00] | 10.50 [6.00-20.00] | 0.2745 |

*Abbreviations. GL, guideline; ICU, intensive care unit; IQR, interquartile range [1^st^ quartile – 3^rd^ quartile]; ROSC, return of spontaneous circulation; WLST, withholding and withdrawing life-sustaining treatment.*

*† p <0.05, comparison vs “Cohort 1” (adjusted for multiple comparison, Dunnett’s test). § p <0.05, Cochran Armitage test for trend.*

**Table S7.** Demographic, anamnestic, and cardiac arrest/resuscitation characteristics of the comatose patients (≥24 h), stratified by cohort

|  | **All patients** | **Cohort 1 - GL 2010**  ***(01/2011–12/2015)*** | **Cohort 2 - GL 2015**  ***(01/2016–03/2021)*** | **Cohort 3 - GL 2021**  ***(04/2021–09/2024)*** | **p-value** |
| --- | --- | --- | --- | --- | --- |
| **Demographic characteristics** | | | | | |
| Patients, n (%) | 221 (100) | 68 (30.77) | 86 (38.91) | 67 (30.32) | - |
| Male sex**^§^**, n (%) | 170 (76.92) | 46 (67.65) | 63 (73.26) | 61 (91.04) ^†^ | 0.0032 |
| Age (years), median [IQR] | 60.00 [50.00-70.00] | 63.50 [54.00-73.00] | 60.00 [50.00-71.00] | 59.00 [51.00-68.00] | 0.2991 |
| CCI (score), median [IQR] | 3.00 [1.00-5.00] | 3.00 [1.00-5.00] | 3.00 [1.00-5.00] | 2.00 [1.00-4.00] | 0.1417 |
| **CA and CPR characteristics** | | | | | |
| OHCA, n (%) | 147 (66.52) | 47 (69.12) | 51 (59.30) | 49 (73.13) | 0.1709 |
| Pathogenesis, n (%) |  |  |  |  |  |
| Medical | 188 (85.07) | 59 (86.76) | 70 (81.40) | 59 (88.06) | 0.4631 |
| Cardiac aetiology **^§^** | 139 (74.33) | 41 (70.69) | 46 (65.71) | 52 (88.14) ^†^ | 0.0110 |
| Traumatic | 6 (2.71) | 1 (1.47) | 4 (4.65) | 1 (1.49) | 0.5130 |
| Drug overdose | 11 (4.98) | 0 (0) | 8 (9.30) | 3 (4.48) | 0.0201 |
| Drowning | 0 (0) | 0 (0) | 0 (0) | 0 (0) | - |
| Electrocution | 0 (0) | 0 (0) | 0 (0) | 0 (0) | - |
| Asphyxia | 16 (7.24) | 8 (11.76) | 4 (4.65) | 4 (5.97) | 0.2657 |
| Shockable rhythm **^§^**, n (%) | 101 (45.70) | 25 (36.76) | 33 (38.37) | 43 (64.18) ^†^ | 0.0013 |
| Non-shockable rhythm **^§^**, n (%) | 120 (54.30) | 43 (63.24) | 53 (61.63) | 24 (35.82) ^†^ | 0.0013 |
| Witnessed cardiac arrest, n (%) | 203 (91.86) | 62 (91.18) | 81 (94.19) | 60 (89.55) | 0.5651 |
| Lay Bystander CPR, n (%) | 85 (38.46) | 25 (36.76) | 29 (33.72) | 31 (46.27) | 0.2692 |
| No flow (min), median [IQR] | 0.00 [0.00-3.00] | 0.00 [0.00-0.00] | 0.00 [0.00-5.00] ^†^ | 0.00 [0.00-3.00] | 0.0233 |
| Low flow (min), median [IQR] | 18.00 [9.50-30.00] | 17.00 [9.00-25.00] | 16.00 [8.00-30.00] | 20.00 [12.00-34.00] | 0.2636 |
| Mechanical CC **^§^**, n (%) | 64 (28.96) | 2 (2.94) | 40 (46.51) ^†^ | 22 (32.84) ^†^ | <.0001 |
| Epinephrine dose (mg), median [IQR] | 2.00 [1.00-4.00] | 2.00 [1.00-4.00] | 3.00 [1.00-4.00] | 2.00 [1.00-5.00] | 0.6873 |
| Amiodarone use, n (%) | 61 (27.73) | 16 (23.88) | 13 (15.12) | 32 (47.76) ^†^ | <.0001 |
| Defibrillation attempts, median [IQR] | 1.00 [0.00-3.00] | 0.00 [0.00-3.00] | 0.00 [0.00-2.00] | 2.00 [0.00-4.00] ^†^ | 0.0076 |
| Patients with comorbidities, n (%) | 186 (84.16) | 54 (79.41) | 74 (86.05) | 58 (86.57) | 0.4336 |
| Patients with active drug treatment, n (%) | 128 (57.92) | 33 (48.53) | 56 (62.12) | 39 (58.21) | 0.1171 |
| SOFA score, median [IQR] | 9.00 [7.00-11.00] | 8.00 [7.00-11.00] | 9.00 [7.00-11.00] | 10.00 [7.00-11.00] | 0.8813 |

*Abbreviations. CA, cardiac arrest; CC, Chest Compression; CCI, Charlson’s comorbidity index; CPR, cardiopulmonary resuscitation; IQR, interquartile range [1^st^ quartile – 3^rd^ quartile]; OHCA, out-of-hospital CA; SOFA, sequential organ failure assessment. † p <0.05, comparison vs “Cohort 1” (adjusted for multiple comparison, Dunnett’s test). § p <0.05, Cochran Armitage test for trend*

**Table S8.** Number of patients with adherence to guidelines by macro-area and single item

|  | **All patients**  (n=221) | **Cohort 1**  **GL 2010**  ***(01/2011–12/2015)***  (n=68) | **Cohort 2**  **GL 2015**  ***(01/2016–03/2021)***  (n=86) | **Cohort 3**  **GL 2021**  ***(04/2021–09/2024)***  (n=67) | **p-value** |  |
| --- | --- | --- | --- | --- | --- | --- |
| **Coronary angiography** | | | | | | |
| Overall adherence | 72 (92.31) | 18 (94.74) | 21 (91.30) | 33 (91.67) | 1.0000 |  |
| **Haemodynamics** | | | | | | |
| Overall adherence **^§^** | 103 (51.50) | 16 (32.00) | 36 (42.86) | 51 (77.27) ^†^ | <.0001 |  |
| MAP | 124 (82.12) | *na* | 70 (82.35) | 54 (81.82) | 0.9322 |  |
| Urinary output**^§^** | 113 (56.22) | 18 (36.00) | 36 (42.86) | 59 (88.06) ^†^ | <.0001 |  |
| Lactate **^§^** | 200 (93.02) | 55 (87.30) | 81 (94.19) | 64 (96.97) | 0.1065 |  |
| **Ventilation** | | | | | | |
| Overall adherence **^§^** | 60 (27.91) | 53 (82.81) | 4 (4.71) ^†^ | 3 (4.55) ^†^ | <.0001 |  |
| SaO_2_ **^§^** | 191 (88.84) | 53 (82.81) | 75 (88.24) | 63 (95.45) | 0.0711 |  |
| TV | 111 (73.03) | *na* | 63 (74.12) | 48 (71.64) | 0.7328 |  |
| PEEP **^§^** | 123 (80.92) | *na* | 60 (70.59) | 63 (94.03) | 0.0003 |  |
| PaO_2_ | 19 (12.58) | *na* | 10 (11.76) | 9 (13.64) | 0.7309 |  |
| PaCO_2_ | 61 (40.40) | *na* | 36 (42.35) | 25 (37.88) | 0.5784 |  |
| **Temperature management** | | | | | | |
| Overall adherence **^§^** | 165 (80.10) | 40 (60.61) | 74 (86.05) ^†^ | 51 (94.44) ^†^ | <.0001 |  |
| **General ICU management** | | | | | | |
| Overall adherence **^§^** | 154 (71.30) | 36 (56.25) | 66 (77.65) ^†^ | 52 (77.61) ^†^ | 0.0065 |  |
| Glycaemia **^§^** | 156 (72.22) | 36 (56.25) | 66 (77.65) ^†^ | 54 (80.60) ^†^ | 0.0028 |  |
| DVTP | 65 (97.01) | *na* | *na* | 65 (97.01) | - |  |
| SUP | 67 (100.00) | *na* | *na* | 67 (100.00) | - |  |
| **Multimodal neuroprognostication** *(at 72h from ICU admission)* | | | | | | |
| Overall adherence | 77 (98.72) | *na* | 42 (97.67) | 35 (100.00) | 1.0000 |  |
| Pupillometry | 31 (88.57) | *na* | *na* | 31 (88.57) | - |  |
| Pupillary and corneal reflexes | 78 (100.00) | *na* | 43 (100.00) | 35 (100.00) | - |  |
| SSEP | 54 (69.23) | *na* | 30 (69.77) | 24 (68.57) | 0.9094 |  |
| EEG | 67 (85.90) | *na* | 37 (86.05) | 30 (85.71) | 1.0000 |  |
| NSE | 64 (82.05) | *na* | 33 (76.74) | 31 (88.57) | 0.1758 |  |
| CT or MRI imaging | 65 (83.33) | *na* | 33 (76.74) | 32 (91.43) | 0.0835 |  |
| **Seizures treatment** | | | | | | |
| Overall adherence | 38 (90.48) | *na* | 24 (85.71) | 14 (100) | 0.2829 |  |

*Note. If the guideline in effect during the period did not include the item, adherence was considered as “not assessable” (na).*

*Abbreviations. CT, computed tomography; DVTP, deep venous thrombosis prophylaxis; EEG, electroencephalogram; GL, guideline; ICU, intensive care unit; MAP, mean arterial pressure; MRI, magnetic resonance imaging; NSE, neurological status evaluation; PaO_2_, arterial oxygen pressure; PaCO_2_, arterial carbon dioxide pressure; PEEP, positive end expiratory pressure; SaO_2_, arterial oxygen saturation; SSEP, somatosensory evoked potential; SUP, stress ulcer prophylaxis; TTM/TC, targeted temperature management/controlled hypothermia; TV, tidal volume.*

*† p <0.05, comparison vs “Cohort 1” (adjusted for multiple comparison, Dunnett’s test). § p <0.05, Cochran Armitage test for trend*

**Table S9.** Predictors for survival and favourable neurological outcome at ICU discharge: mixed-effects models with year as a random effect.

|  | **Coefficient ± SE** | **p-value** | **Odds Ratio (95%CI)** | |  |
| --- | --- | --- | --- | --- | --- |
| **ICU survival** | | | | |  |
| Shockable rhythm (ref. No) | 2.340 ± 0.479 | <.0001 | 10.386 (4.244 - 28.065) | | |
| Adherence – Hemodynamic (ref. No) | 0.786 ± 0.398 | 0.0482 | 2.195 (1.013 - 4.863) | |  |
| Male sex (ref. Female) | 0.005 ± 0.469 | 0.9915 | 1.005 (0.398 - 2.537) | |  |
| Any comorbidity (ref. No) | -0.181 ± 0.615 | 0.7690 | 0.835 (0.245 - 2.778) | |  |
| Time No-Flow (1 minute) | -0.113 ± 0.054 | 0.0363 | 0.893 (0.798 - 0.983) | |  |
| Time Low-Flow (1 minute) | -0.035 ± 0.017 | 0.0358 | 0.965 (0.931 - 0.996) | |  |
| Out-of-hospital CA (ref. In-hospital CA) | -1.241 ± 0.522 | 0.0174 | 0.289 (0.100 - 0.783) | |  |
| Age (1 year) | -0.065 ± 0.017 | 0.0001 | 0.937 (0.905 - 0.967) | |  |
| Epinephrine dose (1 mg) | -0.181 ± 0.092 | 0.0486 | 0.834 (0.692 - 0.995) | |  |
| **Favourable neurological outcome at ICU discharge** | | | |  |  |
| Shockable rhythm (ref. No) | 2.396 ± 0.515 | <.0001 | 10.981 (4.006 - 30.102) | |  |
| Adherence – Hemodynamic (ref. No) | 0.566 ± 0.416 | 0.1734 | 1.762 (0.780 - 3.980) | |  |
| Time No-Flow (1 minute) | -0.060 ± 0.053 | 0.2672 | 0.942 (0.847 - 1.047) | |  |
| GCS=3 at hospital admission (ref. >3) | -1.055 ± 0.481 | 0.0282 | 0.348 (0.136 - 0.893) | |  |
| Age (1 year) | -0.047 ± 0.015 | 0.0020 | 0.954 (0.926 - 0.983) | |  |
| Time Low-Flow (1 minute) | -0.033 ± 0.019 | 0.0834 | 0.968 (0.933 - 1.004) | |  |
| Out-of-hospital CA (ref. in-hospital CA) | -1.753 ± 0.570 | 0.0021 | 0.173 (0.057 - 0.529) | |  |
| Epinephrine dose (1 mg) | -0.218 ± 0.098 | 0.0265 | 0.804 (0.663 - 0.975) | |  |

*Abbreviations. CA, cardiac arrest; CI, confidence interval; GCS, Glasgow coma scale; ICU, intensive care unit; SE, standard error.*

*Note. Both models were performed on 185 patients without missing values in predictors.*

**Table S10.** STROBE Statement—Checklist of items that should be included in reports of cohort studies.

|  | Item No | Recommendation | Page (No) |
| --- | --- | --- | --- |
| **Title and abstract** |  |  |  |
|  | 1 | (*a*) Indicate the study’s design with a commonly used term in the title or the abstract | Abstract (2) |
|  |  | (*b*) Provide in the abstract an informative and balanced summary of what was done and what was found |  |
| Introduction | | | |
| Background/rationale | 2 | Explain the scientific background and rationale for the investigation being reported | 3 – 4 |
| Objectives | 3 | State specific objectives, including any prespecified hypotheses | 4 – 5 |
| Methods | | | |
| Study design | 4 | Present key elements of study design early in the paper | 4 |
| Setting | 5 | Describe the setting, locations, and relevant dates, including periods of recruitment, exposure, follow-up, and data collection | 4 |
| Participants | 6 | (*a*) Give the eligibility criteria, and the sources and methods of selection of participants. Describe methods of follow-up | 4, Figure 1a |
|  |  | (*b*) For matched studies, give matching criteria and number of exposed and unexposed |  |
| Variables | 7 | Clearly define all outcomes, exposures, predictors, potential confounders, and effect modifiers. Give diagnostic criteria, if applicable | 4 – 7, Figure 1b |
| Data sources/ measurement | 8* | For each variable of interest, give sources of data and details of methods of assessment (measurement). Describe comparability of assessment methods if there is more than one group | 4 – 7 |
| Bias | 9 | Describe any efforts to address potential sources of bias | 5 |
| Study size | 10 | Explain how the study size was arrived at | 8 |
| Quantitative variables | 11 | Explain how quantitative variables were handled in the analyses. If applicable, describe which groupings were chosen and why | 8 – 9 |
| Statistical methods | 12 | (*a*) Describe all statistical methods, including those used to control for confounding | 8 – 9 |
|  |  | (*b*) Describe any methods used to examine subgroups and interactions |  |
|  |  | (*c*) Explain how missing data were addressed |  |
|  |  | (*d*) If applicable, explain how loss to follow-up was addressed |  |
|  |  | (*e*) Describe any sensitivity analyses |  |
| Results | | |  |
| Participants | 13* | (a) Report numbers of individuals at each stage of study—eg numbers potentially eligible, examined for eligibility, confirmed eligible, included in the study, completing follow-up, and analysed | 10  Figure 1a |
|  |  | (b) Give reasons for non-participation at each stage |  |
|  |  | (c) Consider use of a flow diagram |  |
| Descriptive data | 14* | (a) Give characteristics of study participants (eg demographic, clinical, social) and information on exposures and potential confounders | (a) Tables 2, S2 – S5, S7 |
|  |  | (b) Indicate number of participants with missing data for each variable of interest | (b) Table S1 |
|  |  | (c) Summarise follow-up time (eg, average and total amount) | (c) Table 4, S6 |
| Outcome data | 15* | Report numbers of outcome events or summary measures over time | Table 4, S6 |
| Main results | 16 | (a) Give unadjusted estimates and, if applicable, confounder-adjusted estimates and their precision (eg, 95% confidence interval). Make clear which confounders were adjusted for and why they were included | Figure 2b – 5  Table 5, Table S9, Figure 4-S1 |
|  |  | (b) Report category boundaries when continuous variables were categorized | - |
|  |  | (c) If relevant, consider translating estimates of relative risk into absolute risk for a meaningful time period | - |
| **Discussion** |  |  |  |
| Key results | 18 | Summarise key results with reference to study objectives | 13 – 15 |
| Limitations | 19 | Discuss limitations of the study, taking into account sources of potential bias or imprecision. Discuss both direction and magnitude of any potential bias | 15 – 16 |
| Interpretation | 20 | Give a cautious overall interpretation of results considering objectives, limitations, multiplicity of analyses, results from similar studies, and other relevant evidence | 13 – 16 |
| Generalisability | 21 | Discuss the generalisability (external validity) of the study results | 15 |
| **Other information** |  |  |  |
| Funding | 22 | Give the source of funding and the role of the funders for the present study and, if applicable, for the original study on which the present article is based | 17 |

*Give information separately for exposed and unexposed groups.

An Explanation and Elaboration article discusses each checklist item and gives methodological background and published examples of transparent reporting. The STROBE checklist is best used in conjunction with this article (freely available on the Web sites of PLoS Medicine at http://www.plosmedicine.org/, Annals of Internal Medicine at http://www.annals.org/, and Epidemiology at http://www.epidem.com/). Information on the STROBE Initiative is available at http://www.strobe-statement.org.
